# Deep Learning Enables Fast and Accurate Imputation of Gene Expression

**DOI:** 10.3389/fgene.2021.624128

**Published:** 2021-04-13

**Authors:** Ramon Viñas, Tiago Azevedo, Eric R. Gamazon, Pietro Liò

**Affiliations:** ^1^Department of Computer Science and Technology, University of Cambridge, Cambridge, United Kingdom; ^2^Vanderbilt Genetics Institute and Data Science Institute, VUMC, Nashville, TN, United States; ^3^MRC Epidemiology Unit, University of Cambridge, Cambridge, United Kingdom; ^4^Clare Hall, University of Cambridge, Cambridge, United Kingdom

**Keywords:** gene expression, transcriptomics, imputation, generative adversarial networks, machine learning, RNA-seq, GTEx, deep learning

## Abstract

A question of fundamental biological significance is to what extent the expression of a subset of genes can be used to recover the full transcriptome, with important implications for biological discovery and clinical application. To address this challenge, we propose two novel deep learning methods, PMI and GAIN-GTEx, for gene expression imputation. In order to increase the applicability of our approach, we leverage data from GTEx v8, a reference resource that has generated a comprehensive collection of transcriptomes from a diverse set of human tissues. We show that our approaches compare favorably to several standard and state-of-the-art imputation methods in terms of predictive performance and runtime in two case studies and two imputation scenarios. In comparison conducted on the protein-coding genes, PMI attains the highest performance in inductive imputation whereas GAIN-GTEx outperforms the other methods in in-place imputation. Furthermore, our results indicate strong generalization on RNA-Seq data from 3 cancer types across varying levels of missingness. Our work can facilitate a cost-effective integration of large-scale RNA biorepositories into genomic studies of disease, with high applicability across diverse tissue types.

## 1. Introduction

High-throughput profiling of the transcriptome has revolutionized discovery methods in the biological sciences. The resulting gene expression measurements can be used to uncover disease mechanisms (Emilsson et al., 2008; Cookson et al., 2009; Gamazon et al., 2018), propose novel drug targets (Evans and Relling, 2004; Sirota et al., 2011), provide a basis for comparative genomics (King and Wilson, 1975; Colbran et al., 2019), and motivate a wide range of fundamental biological problems. In parallel, methods that learn to represent the expression manifold can improve our mechanistic understanding of complex traits, with potential methodological and technological applications, including organs-on-chips (Low et al., 2020) and synthetic biology (Gupta and Zou, 2019), and the integration of heterogeneous transcriptomics datasets.

A question of fundamental biological significance is to what extent the expression of a subset of genes can be used to recover the full transcriptome with minimal reconstruction error. Genes that participate in similar biological processes or that have shared molecular function are likely to have similar expression profiles (Zhang and Horvath, 2005), prompting the question of gene expression prediction from a minimal subset of genes. Moreover, gene expression measurements may suffer from unreliable values because some regions of the genome are extremely challenging to interrogate due to high genomic complexity or sequence homology (Conesa et al., 2016), further highlighting the need for accurate imputation. Moreover, most gene expression studies continue to be performed with specimens derived from peripheral blood or a convenient surrogate (e.g., lymphoblastoid cell lines; LCLs) due to the difficulty of collecting some tissues. However, gene expression may be tissue or cell-type specific, potentially limiting the utility of a proxy tissue.

The missing data problem can adversely affect downstream gene expression analysis. The simple approach of excluding samples with missing data from the analysis can lead to a substantial loss in statistical power. Dimensionality reduction approaches such as principal component analysis (PCA) and singular value decomposition (SVD) (Wall et al., 2003) cannot be applied to gene expression data with missing values. Clustering methods, a mainstay of genomics, such as *k*-means and hierarchical clustering may become unstable even with a few missing values (Troyanskaya et al., 2001).

To address these challenges, we develop two deep learning approaches to gene expression imputation. In both cases, we present an architecture that recovers missing expression data for multiple tissue types under different levels of missingness. In contrast to existing linear methods for deconfounding gene expression (Øystein Sørensen et al., 2018), our methods integrate covariates (global determinants of gene expression; Stegle et al., 2012) to account for their non-linear effects. In particular, a characteristic feature of our architectures is the use of word embeddings (Mikolov et al., 2013) to learn rich and distributed representations for the tissue types and other covariates. To enlarge the possibility and scale of a study's expression data (e.g., by including samples from highly inaccessible tissues), we train our model on RNA-Seq data from the Genotype-Tissue Expression (GTEx) project (The GTEx Consortium, 2015; GTEx Consortium, 2017), a reference resource (v8) that has generated a comprehensive collection of human transcriptome data in a diverse set of tissues.

We show that the proposed approaches compare favorably to several standard and state-of-the-art imputation methods in terms of predictive performance and runtime. In performance comparison on the protein-coding genes, GAIN-GTEx outperforms all the other methods in in-place imputation while PMI displays the highest performance in inductive imputation. Furthermore, we demonstrate that our methods are highly applicable across diverse tissues and varying levels of missingness. Finally, to analyse the cross-study relevance of our approach, we perform imputation on gene expression data from The Cancer Genome Atlas (TCGA; Weinstein et al., 2013) and show that our approach is robust when applied to independent RNA-Seq data.

## 2. Methods

In this section, we introduce two deep learning approaches for gene expression imputation with broad applicability, allowing us to investigate their strengths and weaknesses in several realistic scenarios. Throughout the remainder of the paper, we use script letters to denote sets (e.g., D), upper-case bold symbols to denote matrices or random variables (e.g., **X**), and lower-case bold symbols to denote column vectors (e.g., **x** or ${\bar{\text{q}}}_{j}$). The rest of the symbols (e.g., ${\bar{q}}_{jk}$, *G*, or *f*) denote scalar values or functions.

### 2.1. Problem Formulation

Consider a dataset $D=\left\{\left(\tilde{\text{x}},\text{m},\text{r},\text{q}\right)\right\}$, where $\tilde{\text{x}}\in {\mathbb{R}}^{n}$ represents a vector of gene expression values with missing components; **m** ∈ {0, 1}^*n*^ is a mask indicating which components of the original vector of expression values **x** are missing or observed; *n* is the number of genes; and **q** ∈ ℕ^*c*^ and **r** ∈ ℝ^*k*^ are vectors of *c* categorical (e.g., tissue type or sex) and *k* quantitative covariates (e.g., age), respectively. Our goal is to recover the original gene expression vector **x** ∈ ℝ^*n*^ by modeling the conditional probability distribution $P\left(\text{X}=\text{x}|\tilde{\text{X}}=\tilde{\text{x}},\text{M}=\text{m},\text{R}=\text{r},\text{Q}=\text{q}\right)$, where the upper-case symbols denote the corresponding random variables.

### 2.2. Pseudo-Mask Imputation

We first introduce a novel imputation method named Pseudo-Mask Imputer (PMI).

**Formulation**. Let $\tilde{\text{x}}=\text{m}⊙\text{x}\in {\mathbb{R}}^{n}$ be a vector of gene expression values whose missing components are indicated by a mask vector **m** ∈ {0, 1}^*n*^. Our model is a function *f* : ℝ^*n*^ × {0, 1}^*n*^ × ℝ^*k*^ × ℕ^*c*^ → ℝ^*n*^ that imputes the missing expression values (**1** − **m**) ⊙ **x** as follows:

(1) $$\begin{array}{l} \bar{\text{x}}=f\left(\tilde{\text{x}},\text{m},\text{r},\text{q}\right). \end{array}$$

Here ⊙ denotes element-wise multiplication. The recovered vector of gene expression values is then given by $\text{m}⊙\tilde{\text{x}}+\left(\text{1}-\text{m}\right)⊙\bar{\text{x}}$.

**Optimization**. We optimize the model to maximize the imputation performance on a dynamic subset of observed, *pseudo-missing* components. In particular, we first generate a *pseudo-mask* $\tilde{\text{m}}$ as follows:

(2) $$\begin{array}{l} \tilde{\text{m}}=\text{m}⊙\text{b}\;\text{b}\sim B\left(1,p\right)\;p\sim U\left(\alpha ,\beta \right), \end{array}$$

where **b** ∈ {0, 1}^*n*^ is a vector sampled from a Bernoulli distribution *B* and α ∈ [0, 1] and β ∈ [α, 1] are hyperparameters that parameterize a uniform distribution *U*. Using the *pseudo-mask* $\tilde{\text{m}}$, we split the observed expression values into a set of *pseudo-observed* components $\tilde{\text{x}}$ and a set of *pseudo-missing* components $\tilde{\text{y}}$:

(3) $$\begin{array}{l} \tilde{\text{x}}=\text{x}⊙\tilde{\text{m}}\;\tilde{\text{y}}=\text{x}⊙\text{m}⊙\left(1-\tilde{\text{m}}\right), \end{array}$$

The imputed components are then given by $\bar{\text{x}}=f\left(\tilde{\text{x}},\tilde{\text{m}},\text{r},\text{q}\right)$. We optimize our model to minimize the mean squared error between the ground truth and the imputed *pseudo-missing* components:

(4) $$\begin{array}{l} L\left(\bar{\text{x}},\tilde{\text{y}},\text{m},\tilde{\text{m}}\right)=\frac{1}{Z}(\text{m}⊙\left(1-\tilde{\text{m}}\right){)}^{⊤}{\left(\bar{\text{x}}-\tilde{\text{y}}\right)}^{2}, \end{array}$$

where $Z=\left(\text{m}⊙\left(\text{1}-\tilde{\text{m}}\right){)}^{⊤}(\text{m}⊙\left(\text{1}-\tilde{\text{m}}\right)\right)$ is a normalization term. We summarize our training algorithm in Algorithm 1.

Importantly, the *pseudo-mask* mechanism generates different sets of *pseudo-observed* components for each input example, effectively enlarging the number of training samples. Specifically, the hyperparameters α and β control the fraction of *pseudo-observed* and *pseudo-missing* components through the probability *p* ~ *U*(α, β). On one hand, a low probability *p* yields sparse *pseudo-observed* vectors $\hat{\text{x}}$, resulting in fast convergence but high bias. On the other hand, a high probability *p* yields denser *pseudo-observed* vectors $\hat{\text{x}}$, resulting in low bias but slower convergence. At inference time, *p* is set to 1 and the *pseudo-mask* $\tilde{\text{m}}$ is equal to the input mask **m**.

**Algorithm 1:** Training algorithm

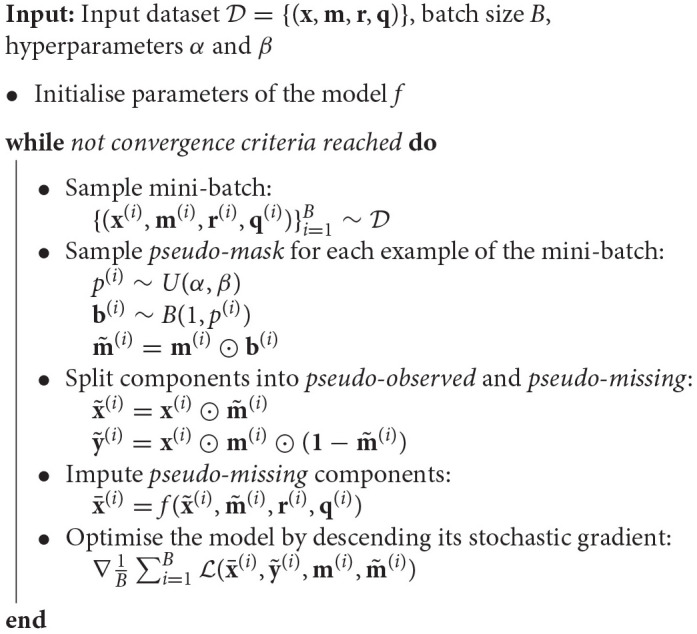

**Architecture**. We model the imputer *f* as a neural network. We first describe how we use word embeddings, a distinctive feature that allows learning rich, dense representations for the different tissue types and, more generally, for all the covariates **q** ∈ ℕ^*c*^.

Formally, let *q*_*j*_ be a categorical covariate (e.g., tissue type) with vocabulary size *v*_*j*_, that is, *q*_*j*_ ∈ {1, 2, …, *v*_*j*_}, where each value in the vocabulary {1, 2, …, *v*_*j*_} represents a different category (e.g., whole blood or kidney). Let ${\bar{\text{q}}}_{j}\in {\left\{0,1\right\}}^{v_{j}}$ be a one-hot vector such that ${\bar{q}}_{jk}=1$ if *q*_*j*_ = *k* and ${\bar{q}}_{jk}=0$ otherwise. Let *d*_*j*_ be the dimensionality of the embeddings for covariate *j*. We obtain a vector of embeddings ${\text{e}}_{j}\in {\mathbb{R}}^{d_{j}}$ as follows:

(5) $$\begin{array}{l} {\text{e}}_{j}={\bar{\text{q}}}_{j}^{⊤}{\text{W}}_{j}, \end{array}$$

where each ${\text{W}}_{j}\in {\mathbb{R}}^{v_{j}\times d_{j}}$ is a matrix of learnable weights. Essentially, this operation describes a lookup search in a dictionary with *v*_*j*_ entries, where each entry contains a learnable *d*_*j*_-dimensional vector of embeddings that characterize each of the possible values that *q*_*j*_ can take. To obtain a global collection of embeddings **e**, we concatenate all the vectors **e**_*j*_ for each categorical covariate *j*:

(6) $$\begin{array}{l} \text{e}={\|}_{j=1}^{c}{\text{e}}_{j}, \end{array}$$

where *c* is the number of categorical covariates and ∥ represents the concatenation operator. We then use the learnable embeddings **e** in downstream tasks.

**Figure 1:**
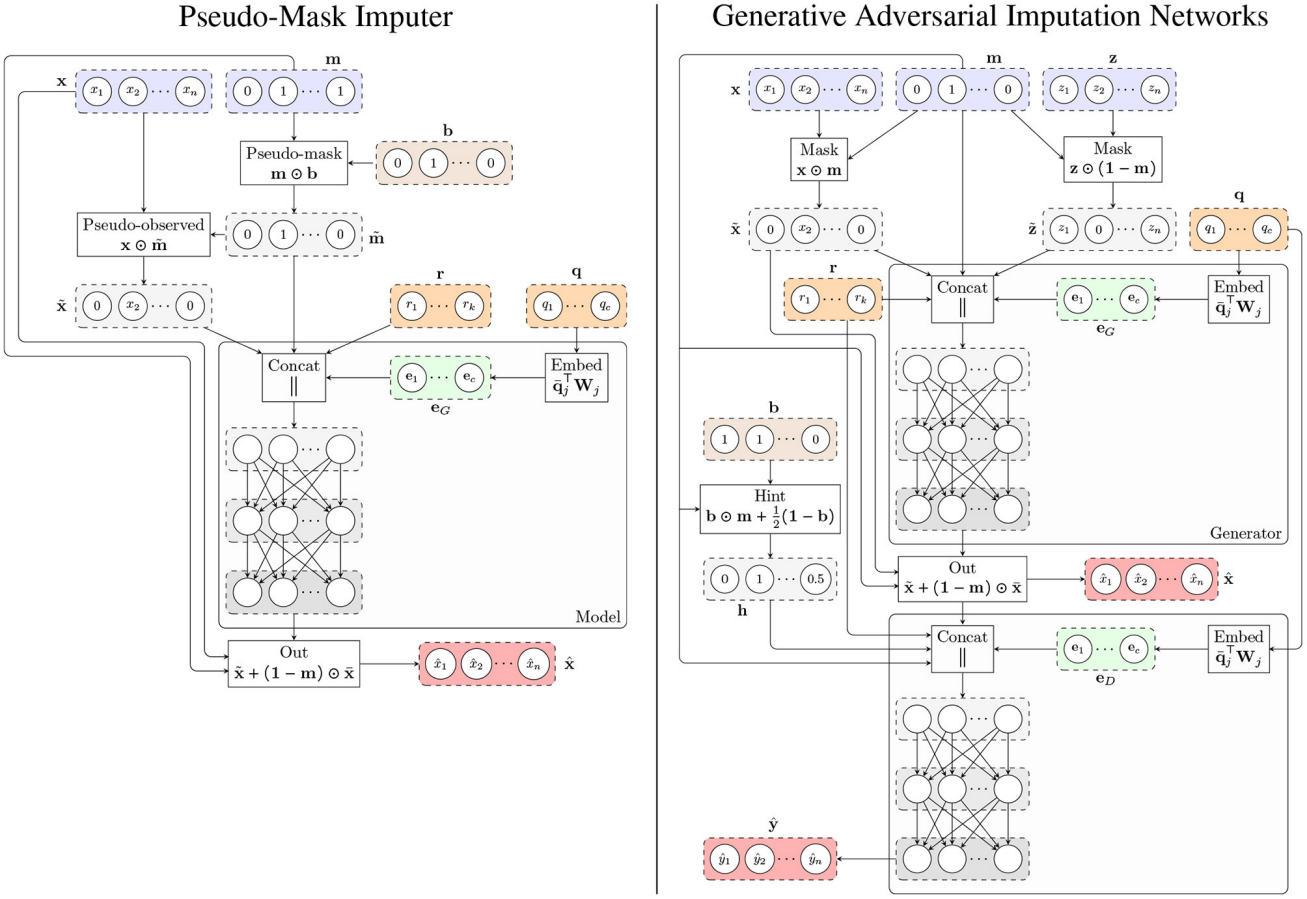
Architecture of the proposed methods. **(Left)** Pseudo-Mask Imputer (PMI). **(Right)** Generative Adversarial Imputation Networks for GTEx (GAIN-GTEx). In both cases, the imputer takes gene expression values $\tilde{\text{x}}$ with missing components according to a mask **m**, and categorical (e.g., tissue type; **q**) and numerical (e.g., age; **r**) covariates, and outputs the imputed values $\bar{\text{x}}$. The observed components of the imputer's output are then replaced by the actual observed expression values $\tilde{\text{x}}$, yielding the imputed sample $\hat{\text{x}}$. For PMI, the *pseudo-mask* mechanism masks out some of the observed components, which are then recovered at the output. For the adversarial model (right), we additionally train a discriminator that receives $\hat{\text{x}}$, the sample covariates, and the hint vector **h**, and produces the output $\hat{\text{y}}$, whose *i*-th component ŷ_*i*_ represents the probability of gene *i* being observed as opposed to being imputed by the generator.

In terms of the architecture, we model *f* as follows:

(7) $$\begin{array}{l} f\left(\tilde{\text{x}},\text{m},\text{r},\text{q}\right)=\text{MLP}\left(\tilde{\text{x}}∥\text{m}∥\text{r}∥\text{e}\right), \end{array}$$

where MLP denotes a multilayer perceptron and $\tilde{\text{x}}=\text{x}⊙\text{m}\in {\mathbb{R}}^{n}$ is the masked gene expression. Figure 1 shows the architecture of the model.

### 2.3. Generative Adversarial Imputation Networks

The second method, which we call GAIN-GTEx, is based on Generative Adversarial Imputation Nets (GAIN; Yoon et al., 2018). Generative Adversarial Networks have previously been used to synthesize transcriptomics *in-silico* (Marouf et al., 2020; Viñas et al., 2021), but to our knowledge their applicability to gene expression imputation is yet to be studied. Similar to generative adversarial networks (GANs; Goodfellow et al., 2014), GAIN estimates a generative model via an adversarial process driven by the competition between two players, the *generator* and the *discriminator*.

**Generator**. The generator aims at recovering missing data from partial gene expression observations, producing samples from the conditional $P\left(\text{X}|\tilde{\text{X}},\text{M},\text{R},\text{Q}\right)$. Formally, we define the generator as a function *G* : ℝ^*n*^ × ℝ^*n*^ × {0, 1}^*n*^ × ℝ^*k*^ × ℕ^*c*^ → ℝ^*n*^ that imputes expression values as follows:

(8) $$\begin{array}{l} \bar{\text{x}}=G\left(\text{x}⊙\text{m},\text{z}⊙\left(1-\text{m}\right),\text{m},\text{r},\text{q}\right), \end{array}$$

where **z** ∈ ℝ^*n*^ is a vector sampled from a fixed noise distribution. Similar to GAIN, we mask the *n*-dimensional noise vector as **z** ⊙ (**1** − **m**), encouraging a bijective association between noise components and genes. Before passing the output $\bar{\text{x}}$ to the discriminator, we replace the prediction for the non-missing components by the original, observed expression values:

(9) $$\begin{array}{l} \hat{\text{x}}=\text{m}⊙\tilde{\text{x}}+\left(1-\text{m}\right)⊙\bar{\text{x}}. \end{array}$$

**Discriminator**. The discriminator takes the imputed samples $\hat{\text{x}}$ and attempts to distinguish whether the expression value of each gene has been observed or produced by the generator. This is in contrast to the original GAN discriminator, which receives information from two input streams (generator and data distribution) and attempts to distinguish the true input source.

Formally, the discriminator is a function *D* : ℝ^*n*^ × ℝ^*n*^ × ℝ^*k*^ × ℕ^*c*^ → ℝ^*n*^ that outputs the probabilities $\hat{\text{y}}\in {\mathbb{R}}^{n}$:

(10) $$\begin{array}{l} \hat{\text{y}}=D\left(\hat{\text{x}},\text{h},\text{r},\text{q}\right), \end{array}$$

where the *i*-th component ŷ_*i*_ is the probability of gene *i* being observed (as opposed to being imputed by the generator) for each *i* ∈ {1, …, *n*} and the vector **h** ∈ ℝ^*n*^ corresponds to the *hint* mechanism described in Yoon et al. (2018), which provides theoretical guarantees on the uniqueness of the global minimum for the estimation of $P\left(\text{X}|\tilde{\text{X}},\text{M},\text{R},\text{Q}\right)$. Concretely, the role of the hint vector **h** is to *leak* some information about the mask **m** to the discriminator. Similar to GAIN, we define the hint **h** as follows:

(11) $$\begin{array}{l} \text{h}=\text{b}⊙\text{m}+\frac{1}{2}\left(1-\text{b}\right)\;\text{b}\sim B\left(1,p\right)\;p\sim U\left(\alpha ,\beta \right), \end{array}$$

where **b** ∈ {0, 1}^*n*^ is a binary vector that controls the amount of information from the mask **m** revealed to the discriminator. In contrast to GAIN, which discloses all but one components of the mask, we sample **b** from a Bernoulli distribution parametrized by a random probability *p* ~ *U*(α, β), where α ∈ [0, 1] and β ∈ [α, 1] are hyperparameters. This accounts for a high number of genes *n* and allows to trade off the number of mask components that are revealed to the discriminator.

**Optimization**. Similarly to GAN and GAIN, we optimize the generator and discriminator adversarially, interleaving gradient updates for the discriminator and generator.

The discriminator aims at determining whether genes have been observed or imputed based on the imputed vector $\hat{\text{x}}$, the covariates **q** and **r**, and the hint vector **h**. Since the hint vector **h** readily provides partial information about the ground truth **m** (Equation 11), we penalize *D* only for genes *i* ∈ {1, 2, …, *n*} such that *h*_*i*_ = 0.5, that is, genes whose corresponding mask value is unavailable to the discriminator. We achieve this via the following loss function $L_{D}:{\left\{0,1\right\}}^{n}\times {\mathbb{R}}^{n}\times {\left\{0,1\right\}}^{n}\to \mathbb{R}$:

(12) $$\begin{array}{l} L_{D}\left(\text{m},\hat{\text{y}},\text{b}\right)=\frac{-1}{Z}{\left(1-\text{b}\right)}^{⊤}(\text{m}⊙\log\hat{\text{y}}+\left(1-\text{m}\right)⊙\left(1-\log\hat{\text{y}}\right)), \end{array}$$

where *Z* = 1 + (**1** − **b**)^⊤^(**1** − **b**) is a normalization term. The only difference with respect to the binary cross entropy loss function is the dot product involving (**1** − **b**), which we employ to ignore genes whose mask has been *leaked* to the discriminator through **h**.

The generator aims at implicitly estimating $P\left(\text{X}|\tilde{\text{X}},\text{M},\text{R},\text{Q}\right)$. Therefore, its role is not only to impute the expression corresponding to missing genes, but also to reconstruct the expression of the observed inputs. Similar to GAIN, in order to account for this and encourage a realistic imputation function, we use the following loss function $L_{G}:{\left\{0,1\right\}}^{n}\times {\mathbb{R}}^{n}\times {\mathbb{R}}^{n}\times {\mathbb{R}}^{n}\times {\left\{0,1\right\}}^{n}\to \mathbb{R}$ for the generator:

(13) $$\begin{array}{l} L_{G}\left(\text{m},\text{x},\bar{\text{x}},\hat{\text{y}},\text{b}\right)=\frac{-1}{Z_{1}}(\left(1-\text{b}\right)⊙\left(1-\text{m}\right){)}^{⊤}\log\hat{\text{y}}+\frac{\lambda }{Z_{2}}{\text{m}}^{⊤}{\left(\text{x}-\bar{\text{x}}\right)}^{2}, \end{array}$$

where $Z_{1}=1+{\left(\text{1}-\text{b}\right)}^{⊤}\left(\text{1}-\text{b}\right)$ and $Z_{2}={\text{m}}^{⊤}\text{m}$ are normalization terms, and λ > 0 is a hyperparameter. Intuitively, the first term in Equation (13) corresponds to the adversarial loss, whereas the mean squared error (MSE) term accounts for the loss that the generator incurs in the reconstruction of the observed gene expression values.

**Architecture**. We model the discriminator *D* and the generator *G* using neural networks. Similar to PMI, *D* and *G* leverage independent instances **e**^*G*^ and **e**^*D*^ of the categorical embeddings described in Equation (6). Specifically, we model the two players as follows:

(14) $$\begin{array}{l} G\left(\tilde{\text{x}},\tilde{\text{z}},\text{m},\text{r},\text{q}\right)=\text{MLP}\left(\tilde{\text{x}}∥\tilde{\text{z}}∥\text{m}∥\text{r}∥{\text{e}}^{G}\right)\\ \;D\left(\hat{\text{x}},\text{h},\text{r},\text{q}\right)=\text{MLP}\left(\hat{\text{x}}∥\text{h}∥\text{r}∥{\text{e}}^{D}\right), \end{array}$$

where MLP denotes a multilayer perceptron and $\tilde{\text{x}}=\text{x}⊙\text{m}\in {\mathbb{R}}^{n}$ and $\tilde{\text{z}}=\text{z}⊙\left(\text{1}-\text{m}\right)\in {\mathbb{R}}^{n}$ are the masked gene expression and noise input vectors, respectively. Figure 1 shows the architecture of both players.

## 3. Experimental details

In this section, we provide an overview of the dataset and describe the experimental details, including all the different case studies and imputation scenarios that we considered. We also describe the implementation details of PMI (see Supplementary Figure 6) and GAIN-GTEx (see Supplementary Figure 7).

### 3.1. Materials

**Dataset**. The GTEx dataset is a public genomic resource of genetic effects on the transcriptome across a broad collection of human tissues, enabling linking of these regulatory mechanisms to trait and disease associations (Aguet et al., 2020). Our dataset contained 15,201 RNA-Seq samples collected from 49 tissues of 838 unique donors. We also selected the intersection of all the protein-coding genes among these tissues, yielding 12,557 unique genes. In addition to the expression data, we leveraged metadata about the sample donors, including sex, age, and cohort (post-mortem, surgical, or organ donor).

**Standardization**. A large proportion of gene expression data in public repositories contains normalized values. Thus, imputation in this context has practical utility. Imputing the relative expression levels (in normalized data) vs absolute levels (in non-normalized data) is also biologically meaningful, with important applications, e.g., differential expression analysis (between disease individuals and controls) that is robust to expression outliers. To this end, we normalized the expression data via the standard score, so that the standardized expression values have mean 0 and standard deviation 1 for each gene across all samples.

**Training, validation, and test splits**. To prevent any leakage of information between the training and test sets, we enforced all samples from the same donor to be within the same set. Concretely, we first flipped the GTEx donor identifiers (e.g., 111CU-1826 is flipped to 6281-UC111), we then sorted the reversed identifiers in alphabetical order, and we finally selected a suitable split point, forcing the two sets to be disjoint. After splitting the data, the training set, which we used to train the model, consisted of ~60% of the total samples. The validation set, which we used to optimize the method, consisted of ~20% of the total samples. The test set, on which we evaluated the final performance, contained the remaining ~20% of the data.

### 3.2. Case Studies

We benchmarked the methods on two case studies:

**Case 1: Protein-coding genes**. As a first case study, we selected the intersection of all the protein-coding genes among the 49 GTEx tissues, resulting in a set of 12,557 unique genes. This case study is challenging for imputation methods that are not scalable across the number of input variables.
**Case 2: Genes in a pathway**. We selected a subset of 273 genes from the Alzheimer's disease pathway extracted from the Kyoto Encyclopedia of Genes and Genomes (KEGG; Kanehisa and Goto, 2000). This case study allows to benchmark imputation methods that do not scale well with the number of variables.

### 3.3. Imputation Scenarios

We considered two realistic imputation scenarios:

**Scenario 1: In-place imputation**. Our goal is to impute the missing values of a dataset D={(m⊙x,m,r,q)} without access to the ground truth missing values (**1** − **m**) ⊙ **x**. Importantly, for this scenario we assumed that the data is *missing completely at random* (MCAR; Little and Rubin, 2019), that is, the missingness does not depend on any of the observed nor unobserved variables.
**Scenario 2: Inductive imputation**. Given a training dataset $D_{train}=\left\{\left(\text{x},\text{1},\text{r},\text{q}\right)\right\}$ where all expression values **x** ∈ ℝ^*n*^ are observed, our goal is to impute the missing expression values of an independent test dataset $D_{test}=\left\{\left(\tilde{\text{x}},\text{m},\text{r},\text{q}\right)\right\}$. Methods trained in inductive mode (e.g., on comprehensive datasets such as GTEx) can be used to perform imputation on small, independent datasets where the small number of samples is insufficient to train a model in in-place mode.

### 3.4. Implementation

For both PMI and GAIN-GTEx, we included the donor's age as numerical covariate in **r** and the tissue type, sex and cohort as categorical covariates in **q**. We normalized the numerical variables via the standard score. For each categorical variable *q*_*j*_ ∈ {1, 2, …, *v*_*j*_}, we used the rule of thumb $d_{j}=\left\lfloor \sqrt{v_{j}}\right\rfloor +1$ to set all the dimensions of the categorical embeddings. We used ReLU activations for each hidden layer in the MLP architectures of both PMI and GAIN (see Equations 7 and 14).

We trained both models using the Adam optimizer (Kingma and Ba, 2014). We used batch normalization (Ioffe and Szegedy, 2015) in the hidden layers of the MLPs, which yielded a significant speed-up to the training convergence according to our experiments. We used early stopping with a patience of 30. The rest of parameters for each model, case study, and imputation scenario are presented in the Supplementary Material.

### 3.5. Baseline Methods

We compared PMI and GAIN-GTEx to several baseline methods:

**Common methods of imputation**. We considered two simple gene expression imputation approaches: blood surrogate and median imputation. The use of blood, an easily accessible tissue, as a surrogate for difficult-to-acquire tissues is done in studies of biomarker discovery, diagnostics, and eQTLs, and in the development of model systems (Gamazon et al., 2018; Kim et al., 2020). For blood surrogate imputation, we imputed missing gene expression values in any given tissue with the corresponding values in whole blood for the same donor. For median imputation, we imputed missing values with the median of the observed tissue-specific gene expression computed across donors.

***k*-Nearest Neighbours**. The *k*-Nearest Neighbours (*k*-NN) algorithm is an efficient method that is commonly used for imputation (Beretta and Santaniello, 2016). Here, we leveraged *k*-NN as a baseline for different values of *k*. This model estimates the missing values of a sample based on the values of the missing components in the *k* closest samples.

**State-of-the-art methods**. We considered two state-of-the-art imputation methods: Multivariate Imputation by Chained Equations (MICE; Buuren and Groothuis-Oudshoorn, 2010) and MissForest (Stekhoven and Bühlmann, 2012). MICE leverages chained equations to create multiple imputations of missing data. The hyperparameters of MICE include the minimum/maximum possible imputed value for each component and the maximum number of imputation rounds. MissForest (Stekhoven and Bühlmann, 2012) is a non-parametric imputation method based on random forests trained on observed values to impute the missing values. Among others, the hyperparameters of MissForest include the number of trees in the forest and the number of features to consider when looking for the optimal split.

## 4. Results

Here we provide an overview of the imputation results, including a comparison with other imputation methods, an evaluation of the tissue-specific results, and an analysis of the cross-study relevance across different levels of missingness.

### 4.1. Comparison

Tables 1, 2 show a quantitative summary of the imputation performances for the two case-studies and the two imputation scenarios. In addition to the imputation scores, we report the runtime of all the methods. We labeled methods as computationally *unfeasible* when they took longer than 7 days to run on our server (CPU: Intel(R) Xeon(R) Processor E5-2630 v4. RAM: 125GB), after which we halted the execution. For example, MICE and MissForest were unfeasible for each imputation scenario on the complete set of protein-coding genes. An empirical study of the scalability of both methods (see Supplementary Material) showed that the runtime increases rapidly with the number of genes. However, on a smaller set of genes (i.e., 273 from the Alzheimer's disease pathway), evaluation of the performance was successfully obtained, with the runtime substantially higher for both methods than for the other methods. In addition, we included GAIN-MSE-GTEx as a baseline, consisting of a simplification of GAIN-GTEx that was optimized exclusively via the mean squared error term of the generator. GAIN-MSE-GTEx performed reasonably well relative to GAIN-GTEx, suggesting that the mean squared error term of the loss function was driving the learning (see Supplementary Material).

**Table 1:** Gene expression imputation performance with a missing rate of 50% across 3 runs (complete set of protein-coding genes).

	**Scenario 1: In-place imputation**		**Scenario 2: Inductive imputation**	
**Method**	***R*^2^**	**Runtime (hours)**	***R*^2^**	**Runtime (hours)**
MICE	−	−	−	−
MissForest	−	−	−	−
Blood surrogate	−0.693 ± 0.000	0.000 ± 0.000	−0.952 ± 0.000	0.000 ± 0.000
Median imputation	0.000 ± 0.000	0.001 ± 0.000	−0.009 ± 0.000	0.001 ± 0.000
1-NN imputation	0.179 ± 0.000	1.616 ± 0.004	0.203 ± 0.000	0.985 ± 0.003
5-NN imputation	0.461 ± 0.000	2.224 ± 0.107	0.482 ± 0.000	1.441 ± 0.096
10-NN imputation	0.468 ± 0.000	2.140 ± 0.035	0.495 ± 0.000	1.711 ± 0.160
GAIN-MSE-GTEx	0.637 ± 0.005	0.199 ± 0.074	0.638 ± 0.003	0.456 ± 0.053
GAIN-GTEx	0.638 ± 0.007	0.625 ± 0.294	0.636 ± 0.001	1.199 ± 0.157
PMI	0.479 ± 0.003	0.241 ± 0.024	0.707 ± 0.001	0.244 ± 0.019

*We do not report the R^2^ scores for MICE and MissForest, because the runtime is longer than 7 days. Note GAIN-GTEx outperforms all the other methods in in-place imputation while PMI displays the highest performance in inductive imputation*.

**Table 2:** Gene expression imputation performance with a missing rate of 50% across 3 runs (for a subset of 273 genes from the Alzheimer's disease pathway).

	**Scenario 1: In-place imputation**		**Scenario 2: Inductive imputation**	
**Method**	***R*^2^**	**Runtime (hours)**	***R*^2^**	**Runtime (hours)**
MICE	0.574 ± 0.001	2.062 ± 0.335	0.569 ± 0.001	2.252 ± 0.096
MissForest (1 tree)	−0.147 ± 0.002	0.145 ± 0.002	−0.042 ± 0.003	0.575 ± 0.167
MissForest (10 trees)	0.458 ± 0.001	0.839 ± 0.176	0.514 ± 0.001	3.220 ± 0.371
MissForest (20 trees)	0.478 ± 0.000	1.836 ± 0.068	0.540 ± 0.000	4.842 ± 0.495
MissForest (100 trees)	0.493 ± 0.000	6.438 ± 0.498	0.561 ± 0.001	16.186 ± 1.709
Blood surrogate	−0.698 ± 0.002	0.000 ± 0.000	−0.971 ± 0.002	0.000 ± 0.000
Median imputation	0.001 ± 0.000	0.000 ± 0.000	−0.009 ± 0.000	0.000 ± 0.000
1-NN imputation	0.186 ± 0.001	0.037 ± 0.001	0.301 ± 0.000	0.021 ± 0.001
GAIN-MSE-GTEx	0.519 ± 0.001	0.038 ± 0.002	0.533 ± 0.001	0.045 ± 0.004
GAIN-GTEx	0.533 ± 0.001	0.139 ± 0.041	0.527 ± 0.003	0.569 ± 0.017
PMI	0.536 ± 0.001	0.048 ± 0.002	0.630 ± 0.011	0.037 ± 0.002

In terms of the evaluation metrics, we report the coefficient of determination (*R*^2^). This metric ranges from −∞ to 1 and corresponds to the ratio of explained variance to the total variance. Negative scores indicate that the model predictions are worse than those of a baseline model that predicts the mean of the data. Here, to evaluate the performance, we generated random masks with a missing rate of 50% and computed the imputation *R*^2^ per gene. We repeated the last step 3 times and reported the overall mean *R*^2^ and the average per-gene standard deviation of the *R*^2^ scores, averaged across the 3 runs. In inductive mode, blood surrogate and median imputation exhibited negative scores. Under in-place imputation on the protein-coding genes, GAIN-GTEx outperformed all the other methods (0.638 ± 0.007). Under inductive imputation on the protein-coding genes, PMI showed the best overall performance (0.707 ± 0.001) among all the methods.

### 4.2. Imputation Results

**Figure 2:**
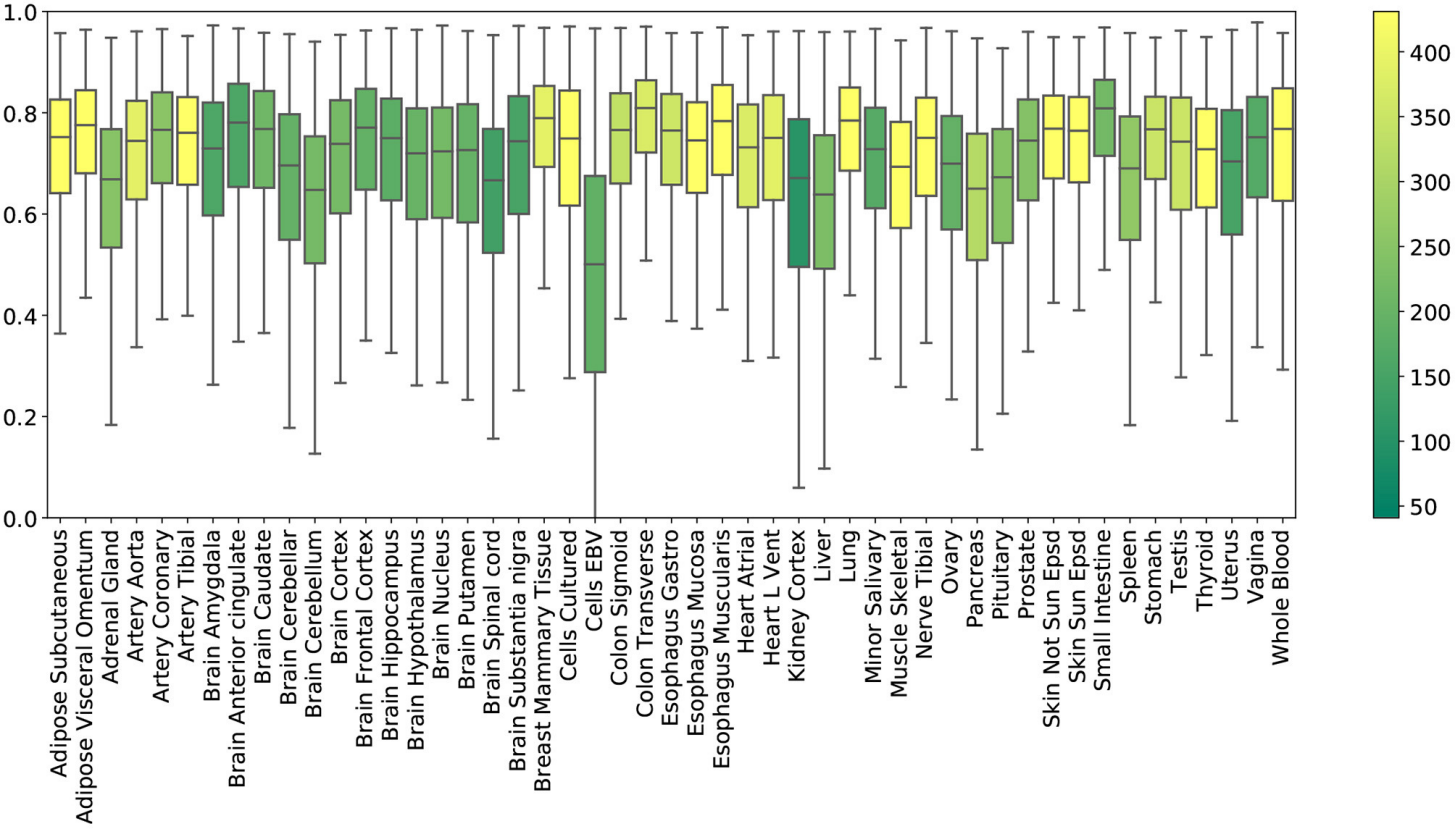
*R*^2^ imputation scores per GTEx tissue with a missing rate of 50% (PMI; inductive mode). Each box shows the distribution of the per-gene *R*^2^ scores in the extended test set. The color of each box represents the number of training samples of the corresponding tissue.

**Tissue-specific results**. Figure 2 shows the *R*^2^ scores achieved by PMI across all 49 tissue types. To obtain these results, we generated random masks with a missing rate of 50% for the test set, performed imputation, and plotted the distribution of 12,557 gene *R*^2^ scores for each tissue. Mean *R*^2^ scores in the individual tissues ranged from ~0.5 (Epstein Barr virus transformed lymphocytes; EBV) to ~0.78 (small intestine). Kidney cortex, the tissue with the smallest sample size, had the highest variability in *R*^2^ with an interquartile range of *Q*_3_ − *Q*_1_ = 0.30.

**Figure 3:**
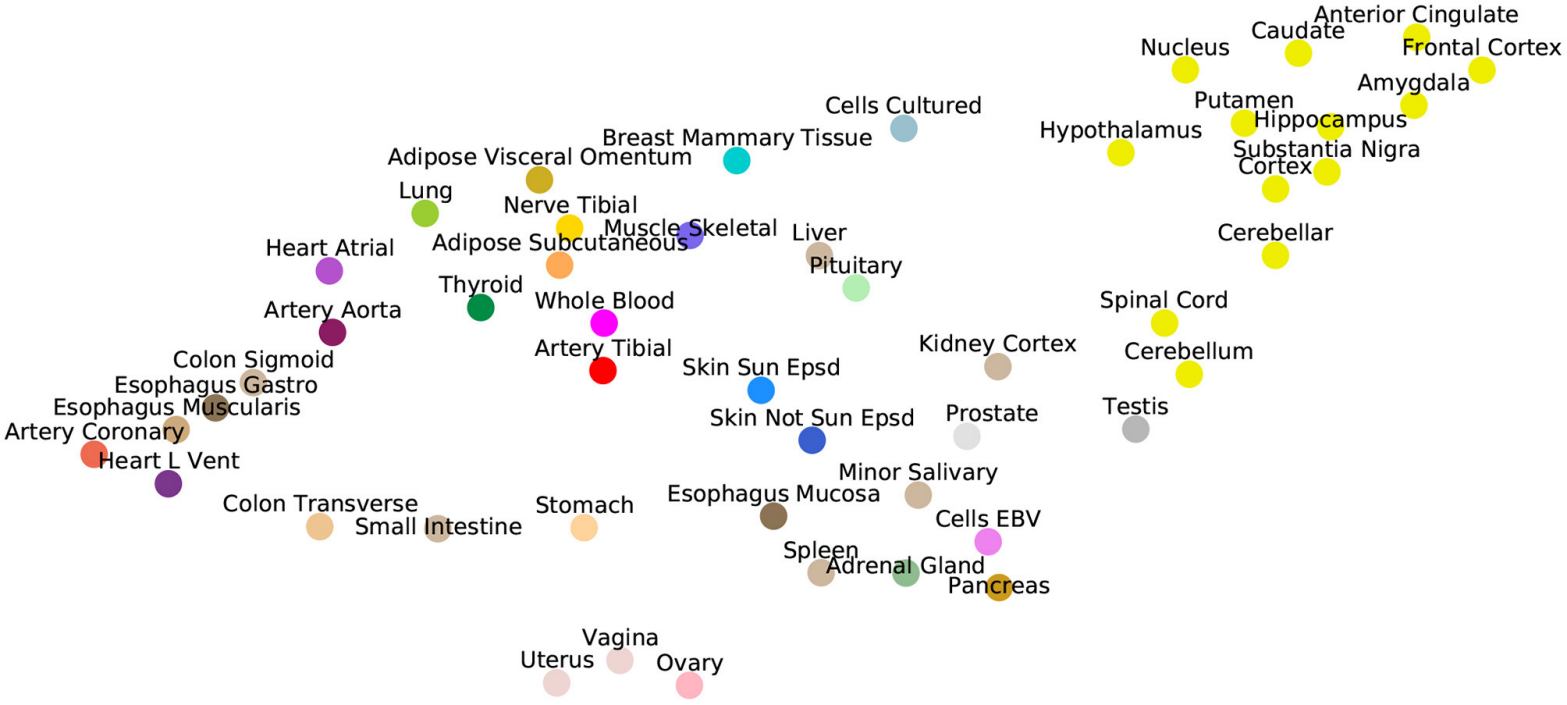
UMAP visualization of the tissue embeddings from the generator. Colors are assigned to conform to the GTEx Consortium conventions. Note that the central nervous system, consisting of the 13 brain regions, clusters together on the top right corner.

Figure 3 illustrates the ability of GAIN-GTEx to learn rich tissue representations. Specifically, we plotted a UMAP representation (McInnes et al., 2018) of the learnt tissue embeddings ${\text{W}}_{j}\in {\mathbb{R}}^{49\times 8}$ from the generator (see Equation 5), where *j* indexes the tissue dimension. Strikingly, the tissue representations showed strong clustering of biologically-related tissues, including the central nervous system (i.e., the 13 brain regions), the gastrointestinal system (e.g., the esophageal and colonic tissues), and the female reproductive tissues (i.e., uterus, vagina, and ovary). The clustering properties were robust across UMAP runs and could be similarly appreciated using other dimensionality reduction algorithms such as tSNE (Maaten and Hinton, 2008).

**Figure 4:**
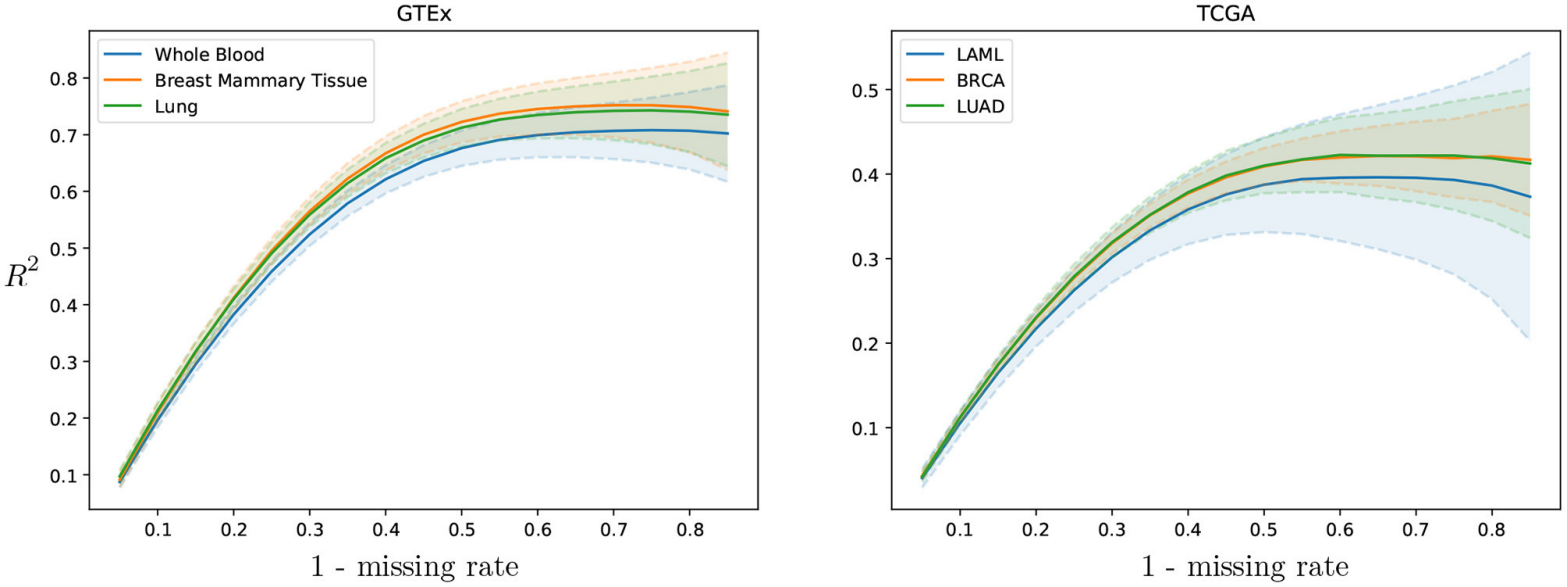
GAIN-GTEx *R*^2^ imputation scores per tissue across missing rate for 3 TCGA cancer types and their healthy counterpart in GTEx. The shaded area represents one standard deviation of the per-gene *R*^2^ scores in the corresponding tissue. The greater the rate of missingness, the lower the performance.

**Cross-study results across missing rates**. To evaluate the cross-study relevance and generalizability of PMI and GAIN-GTEx, we leveraged the model trained on GTEx to perform imputation on The Cancer Genome Atlas (TCGA) gene expression data in acute myeloid leukemia (TCGA LAML; Cancer Genome Atlas Research Network et al., 2013), breast cancer (TCGA BRCA; Cancer Genome Atlas Network, 2012), and lung adenocarcinoma (TCGA LUAD; Cancer Genome Atlas Research Network, 2014). For each TCGA tissue and its *non-diseased* test counterpart on GTEx, we show the imputation quality in Table 3 as well as the performance across varying missing rates in Figure 4.

**Table 3:** Cross-study results for GAIN-GTEx and PMI trained on GTEx (inductive mode).

**GAIN-GTEx**		**PMI**	
**Tissue**	***R*^2^**	**Tissue**	***R*^2^**
TCGA LAML	0.386 ± 0.057	TCGA LAML	0.394 ± 0.065
TCGA BRCA	0.408 ± 0.023	TCGA BRCA	0.427 ± 0.023
TCGA LUAD	0.439 ± 0.034	TCGA LUAD	0.451 ± 0.050
GTEx Whole blood	0.678 ± 0.031	GTEx Whole blood	0.709 ± 0.034
GTEx Breast	0.724 ± 0.036	GTEx Breast	0.751 ± 0.039
GTEx Lung	0.713 ± 0.033	GTEx Lung	0.744 ± 0.035

*We report the R^2^ scores on data from 3 TCGA cancer types and their healthy counterpart on GTEx for a missing rate of 50%*.

**Figure 5:**
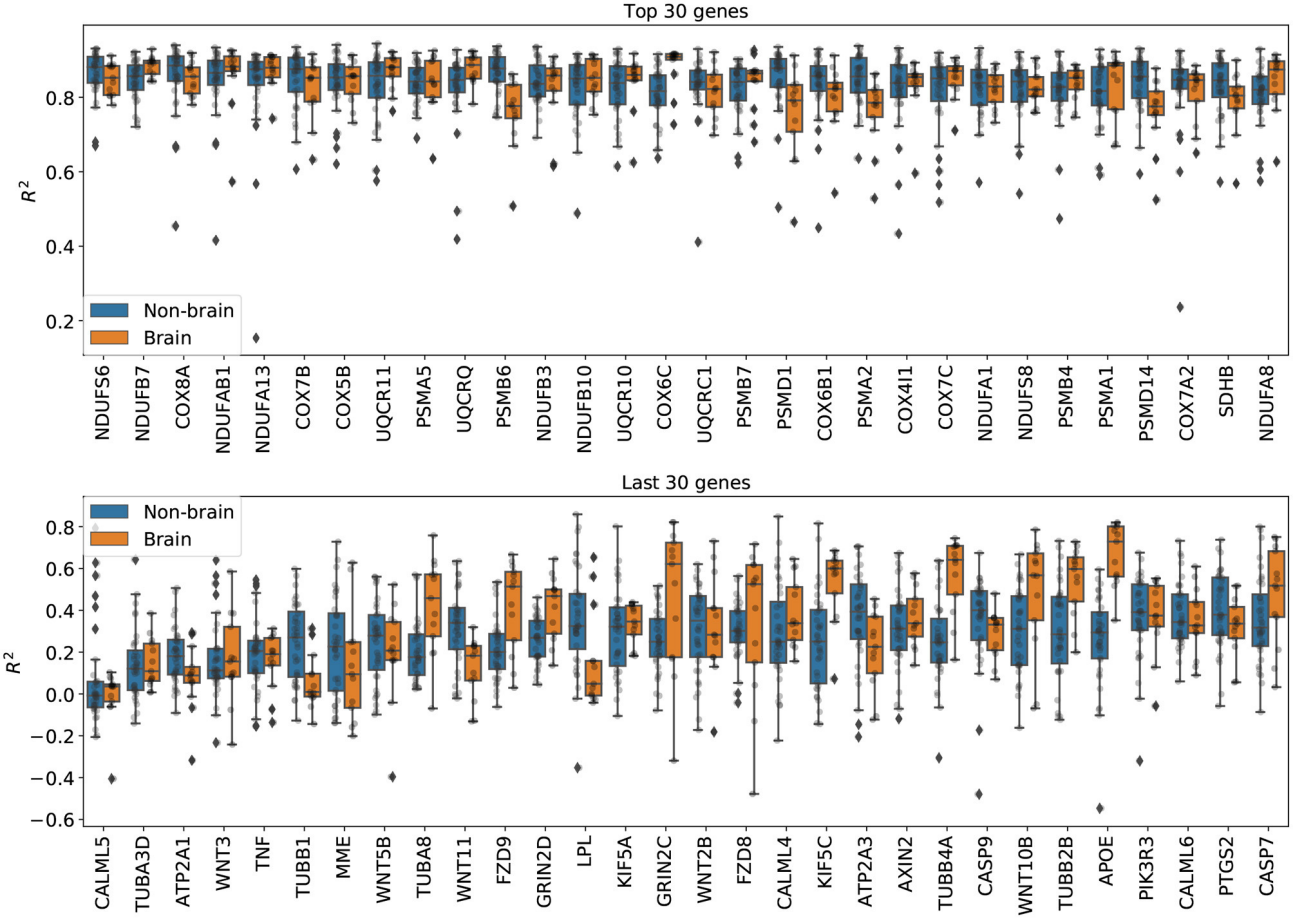
Per-gene imputation *R*^2^ scores on genes from the Alzheimer's disease pathway. Each point represents the average *R*^2^ score in a tissue type. We note that some genes in the pathway (e.g., PSMB6, COX6C, PSMD7, PSMA2, PSMD14, SDHB, TUBB1, TUBA8, FZD9, LPL, KIF5C, TUBB4A, TUBB2B, APOE) exhibited different distributions between brain and non-brain tissue types.

**Imputation results on genes from the Alzheimer's disease pathway**. Figure 5 shows the per-gene imputation scores for GAIN-GTEx trained on a subset of 273 genes corresponding to the Alzheimer's disease pathway. Amyloid-beta is a core element of senile plaques which are characteristic of the debilitating disease, with various pathophysiological consequences on cellular processes. The pathway consists of genes that are involved in a number of processes, including neuronal apoptosis, autophagy deficits, mitochondrial defect, and neurodegeneration. We observed that some genes in the pathway (e.g., PSMB6, COX6C, PSMD7, PSMA2, PSMD14, SDHB, TUBB1, TUBA8, FZD9, LPL, KIF5C, TUBB4A, TUBB2B, APOE) exhibited different distributions between brain and non-brain tissue types. The most highly imputed genes were enriched in known gene sets (see Supplementary Figures 9, 10).

## 5. Discussion

We developed two imputation approaches to gene expression, facilitating the reconstruction of a high-dimensional molecular trait that is central to disease biology and drug target discovery. The proposed methods, which we called Pseudo-Mask Imputer (PMI) and GAIN-GTEx, were able to approximate the gene expression manifold from incomplete gene expression data and relevant covariates (potential global determinants of expression) and impute missing expression values. A characteristic feature of our architectures is the use of word embeddings, which enabled to learn distributed representations of the tissue types (see Figure 3). Importantly, this allowed to condition the imputation algorithms on factors that drive gene expression, endowing the architectures with the ability to represent them in a biologically meaningful way.

We leveraged the most comprehensive human transcriptome resource available (GTEx), allowing us to test the performance of our method in a broad collection of tissues (see Figure 2). The biospecimen repository includes commonly used surrogate tissues (whole blood and EBV transformed lymphocytes), central nervous system tissues from 13 brain regions, and a wide diversity of other primary tissues from *non-diseased* individuals. In particular, we observed that EBV transformed lymphocytes, an accessible and renewable resource for functional genomics, are a notable outlier in imputation performance. This is perhaps not surprising, consistent with studies about the transcriptional effect of EBV infection on the suitability of the cell lines as a model system for primary tissues (Carter et al., 2002). Interestingly, similar tissues exhibit similar *R*^2^ scores (see Supplementary Figure 12).

We analyzed the performance of the proposed approaches and found that they compare favorably to several existing imputation methods in terms of imputation performance and runtime (see Table 1). We observed that standard approaches such as leveraging the expression of missing genes from a surrogate blood tissue yielded negative *R*^2^ values and therefore did not perform well. Median imputation, although easy to implement, had a very limited predictive power. Imputation methods based on *k*-Nearest Neighbours were computationally feasible and yielded solid but poorer *R*^2^ scores. In terms of state-of-the-art-methods, MICE and MissForest were computationally prohibitive given the high-dimensionality of the data and we halted the execution after running our experiments for 7 days. In particular, we performed an empirical study of the scalability of both methods (see Supplementary Figures 1–5) and observed that the runtime increases very rapidly with the number of genes. To alleviate this issue, we compared PMI and GAIN-GTEx with these methods on a subset of 273 genes from the Alzheimer's disease pathway (see Table 2). Under the in-place imputation scenario (Alzheimer's disease pathway), MICE performed better than PMI, GAIN-GTEx, and MissForest (100 trees). Under the inductive imputation setting, PMI outperformed all the other methods by a large margin.

In terms of the comparison between PMI and GAIN-GTEx, our experiments suggest that the latter is generally harder to optimize (see hyperparameter search in Supplementary Material). In particular, GAIN resembles a deep autoencoder in that the supervised loss penalizes the reconstruction error of the observed components. While this is a natural choice, autoencoder-like architectures are considerably sensitive to the user-definable bottleneck dimension. On one hand, a small number of units results in under-fitting. On the other hand, an excessively big bottleneck dimension allows the neural network to trivially *copy-paste* the observed components. In contrast, the loss function of PMI does not penalize the reconstruction error for the *pseudo-observed* components (e.g., the loss function of PMI penalizes the prediction error of the *pseudo-missing* components, which are not provided as input at training time). Together with the fact that the *pseudo-mask* mechanism dynamically enlarges the training size, this subtlety allows training considerably bigger networks without over-fitting. Finally, we observed that a simplification of GAIN-GTEx, GAIN-MSE-GTEx, performed similarly well, suggesting that the mean squared error term of the generator's loss function is driving the learning process. In Supplementary Material, we discuss our empirical findings about the adversarial loss of GAIN. For the purpose of reproducibility, as the gains of the adversarial loss appear to be small or negligible given our observations, we recommend training GAIN-GTEx without the adversarial term.

To evaluate the cross-study relevance of our method, we applied the trained models derived from GTEx (inductive mode) to perform imputation on The Cancer Genome Atlas gene expression data in acute myeloid leukemia, lung adenocarcinoma, and breast cancer. In addition to technical artifacts (e.g., batch effects), generalizing to this data is highly challenging because the expression is largely driven by features of the disease such as chromosomal abnormalities, genomic instabilities, large-scale mutations, and epigenetic changes (Baylin and Jones, 2011; Weinstein et al., 2013). Our results show that, despite these challenges, the methods were robust to gene expression from multiple diseases in different tissues (see Table 3), lending themselves to being used as tools to extend independent transcriptomic studies. Next, we evaluated the imputation performance of PMI and GAIN-GTEx for a range of values for the missing rate (see Figure 4 and Supplementary Figure 8). We noted that the performance is stable and that the greater the proportion of missing values, the lower the prediction performance. Finally, we analyzed the imputation performance across genes from the Alzheimer's disease pathway (see Figure 5) and across all genes (see Supplementary Figure 9). We observed that the most highly imputed imputed genes are non-random and, indeed, cluster in some known pathways (see Supplementary Figures 10, 11).

**Broader Impact**. The study of the transcriptome is fundamental to our understanding of cellular and pathophysiological processes. High-dimensional gene expression data contain information relevant to a wide range of applications, including disease diagnosis (Huang et al., 2010), drug development (Sun et al., 2013), and evolutionary inference (Colbran et al., 2019). Thus, accurate and robust methods for imputation of gene expression have the enormous potential to enhance our molecular understanding of complex diseases, inform the search for novel drugs, and provide key insights into evolutionary processes. Here, we developed a methodology that attains state-of-the art performance in several scenarios in terms of imputation quality and execution time. Our analysis showed that the use of blood as a surrogate for difficult-to-acquire tissues, as commonly practized in biomedical research, may lead to substantially degraded performance, with important implications for biomarker discovery and therapeutic development. Our method generalizes to gene expression in a disease class which has shown considerable health outcome disparities across population groups in terms of morbidity and mortality. Future algorithmic developments therefore hold promise for more effective detection, diagnosis, and treatment (Hosny and Aerts, 2019) and for improved implementation in clinical medicine (Char et al., 2018). Increased availability of transcriptomes in diverse human populations to enlarge our training data (a well-known and critical ethical challenge) should lead to further gains (i.e., decreased biases in results and reduced health disparities) (Wojcik et al., 2019). This work has the potential to catalyze research into the application of deep learning to molecular reconstruction of cellular states and downstream gene mapping of complex traits (Cookson et al., 2009; Zhou et al., 2020).

## 6. Conclusion

In this work, we developed two methods for gene expression imputation, which we named PMI and GAIN-GTEx. To increase the applicability of the proposed methods, we trained them on RNA-Seq data from the Genotype-Tissue Expression project, a reference resource that has generated a comprehensive collection of transcriptomes in a diverse set of tissues. A characteristic feature of our architectures is the use of word embeddings to learn distributed representations for the tissue types. Our approaches compared favorably to several standard and state-of-the-art imputation methods in terms of predictive performance and runtime, and generalized to transcriptomics data from 3 cancer types of the The Cancer Genome Atlas. PMI and GAIN-GTEx show optimal performance among the methods in inductive and in-place imputation, respectively, on the protein-coding genes. This work can facilitate the straightforward integration and cost-effective repurposing of large-scale RNA biorepositories into genomic studies of disease, with high applicability across diverse tissue types.

## Data Availability Statement

The datasets analyzed for this study can be found in the GTEx portal: https://gtexportal.org/. A detailed summary of the GTEx samples and donor information can be found at: https://gtexportal.org/home/tissueSummaryPage. Our code is publicly available at https://github.com/rvinas/GTEx-imputation.

## Ethics Statement

Ethical review and approval was not required for the study on human participants in accordance with the local legislation and institutional requirements. Written informed consent for participation was not required for this study in accordance with the national legislation and the institutional requirements.

## Author Contributions

RV and TA developed and trained the deep learning algorithm, generated all the results and figures. ERG provided the standardized RNA-seq data. ERG and PL supervised the study as joint senior authors. All the authors wrote and approved the manuscript.

## Conflict of Interest

ERG receives an honorarium from the journal *Circulation Research* of the American Heart Association, as a member of the Editorial Board. The remaining authors declare that the research was conducted in the absence of any commercial or financial relationships that could be construed as a potential conflict of interest.
