# Supplementary material for: Deep Learning Enables Fast and Accurate Imputation of Gene Expression
Source: Front Genet. 2021 Apr 13;12:624128. doi: 10.3389/fgene.2021.624128 (PMC8076954; doi:10.3389/fgene.2021.624128)
Supplement: Supplementary file 1 [file Data_Sheet_1.PDF]

Supplementary Material: Deep learning enables fast and accurate imputation of gene expression across tissues

Authors: Ramon Viñas [1,*], Tiago Azevedo [1], Eric R. Gamazon [2,3,4,*], and Pietro Liò [1,*]

[1]Department of Computer Science and Technology, University of Cambridge, Cambridge, UK
[2]Vanderbilt Genetics Institute and Data Science Institute, VUMC, Nashville, TN, USA
[3]MRC Epidemiology Unit, University of Cambridge, Cambridge, UK
[4]Clare Hall, University of Cambridge, Cambridge, UK

* Correspondence to: rv340@cam.ac.uk, ericgamazon@gmail.com, pl219@cam.ac.uk

1 OBSERVATIONS ABOUT GAIN'S ADVERSARIAL LOSS

We have implemented the adversarial loss of Generative Adversarial Imputation Networks (GAIN) as described in the GAIN paper (Yoon et al., 2018). Our implementation can be found at: `https://github.com/rvinas/GAIN-GTEx`.

Our results show that the effects of the adversarial loss on the R^2 imputation scores are small or negligible. We have investigated this issue in great detail and our observations are the following:

- One hypothesis is that the dimensionality of the gene expression data might be too high for GAIN. This was also discussed in a Github issue[1]. For the Alzheimer's disease pathway case study (273 genes) and the in-place scenario, including the adversarial term seems to yield a small improvement in the R^2 scores. Nonetheless, the scores are fairly similar for the other scenarios.
- The weights for the adversarial and mean squared error (MSE) terms might not be properly adjusted. However, when we set the MSE weight to 0, the model failed to converge and the R^2 results were very poor. Without the MSE loss, the training was unstable in all our experiments. Additionally, as described in a Github issue[2], decreasing the weight of the MSE term (e.g., from 1 to 0.1) leads to slower convergence.
- The adversarial loss might be incompatible with certain features of the model or hyperparameter configurations. However, different hyperparameters (including batch normalisation, dropout, and number of hidden units per layer) led to a similar performance with and without adversarial loss.
- The discriminator and generator might need to be well balanced, that is, the discriminator might require more gradient updates to learn useful representations of the data. This idea was also discussed in a Github issue[3], where it is also argued that the model is very sensitive to different hyperparameter configurations. However, after several experiments (e.g., we trained the discriminator more often than the generator), we did not observe significant improvements relative to using the MSE loss exclusively.

For the purpose of reproducibility, as the gains of the adversarial loss appear to be small or negligible given our observations, we recommend training GAIN-GTEx without the adversarial term.

[1] `https://github.com/jsyoon0823/GAIN/issues/9`
[2] `https://github.com/jsyoon0823/GAIN/issues/8`
[3] `https://github.com/jsyoon0823/GAIN/issues/17`

2 SCALABILITY ANALYSIS FOR MISSFOREST

Figures S1 and S2 show the runtime of *a single* iteration of the MissForest algorithm (Stekhoven and Bühlmann, 2012) as we vary the number of genes and samples. We fix the number of trees to 3 and the maximum depth per tree to 3.

Figure S3 shows the runtime of MissForest for a subset as we vary the number of estimators (trees). Importantly, we selected a subset of 273 genes from the Alzheimer's disease pathway and kept all samples.

We kept all the non-specified hyperparameters to their default values. Our implementation is based on Python 3.7.6 and the library `missingpy`. We ran the algorithm with 10 concurrent jobs.

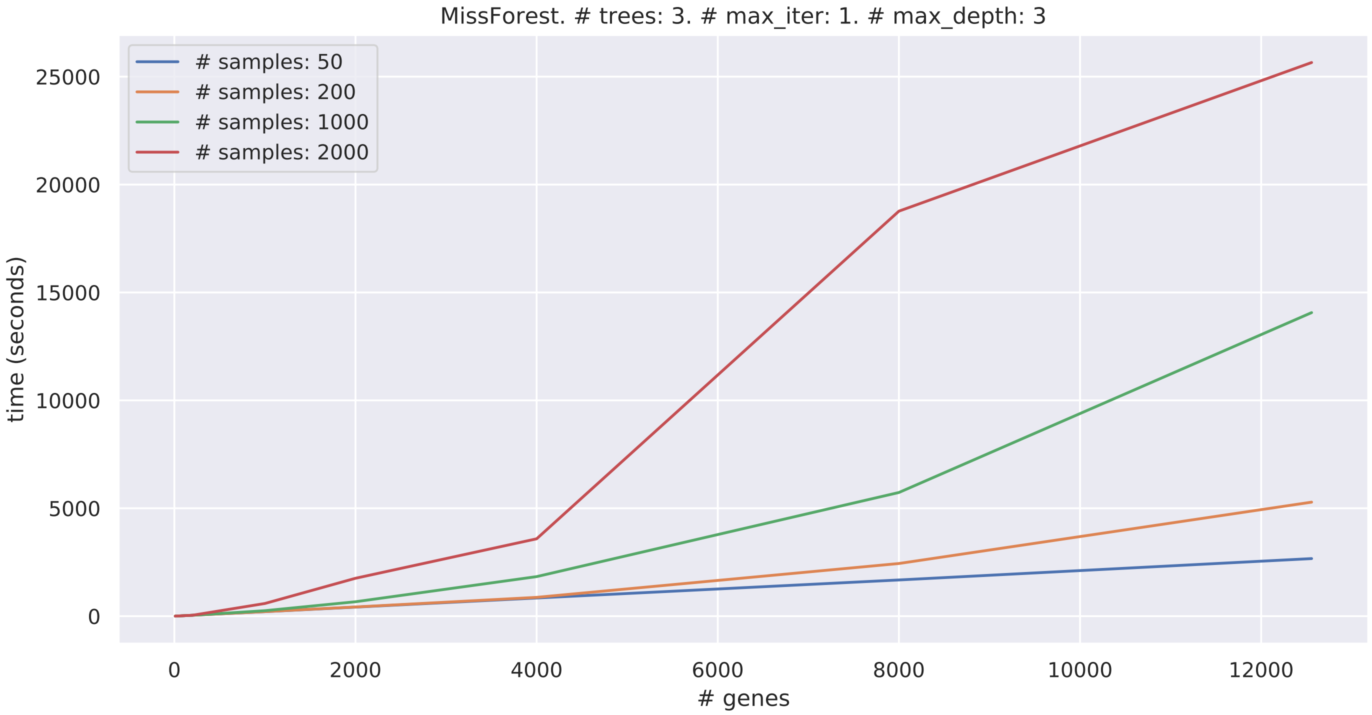

Figure S1: Runtime of *a single* iteration of the MissForest algorithm (Stekhoven and Bühlmann, 2012) as we vary the number of genes.

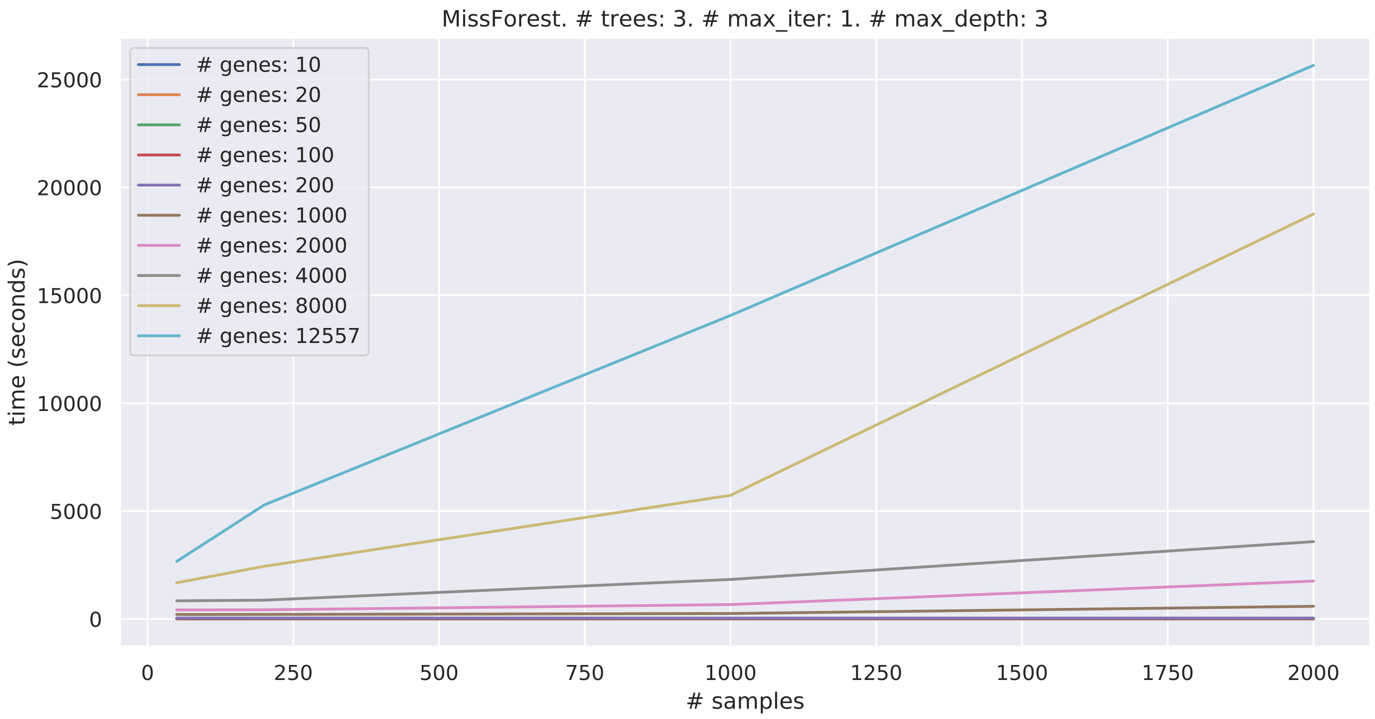

Figure S2: Runtime of *a single* iteration of the MissForest algorithm (Stekhoven and Bühlmann, 2012) as we vary the number of samples.

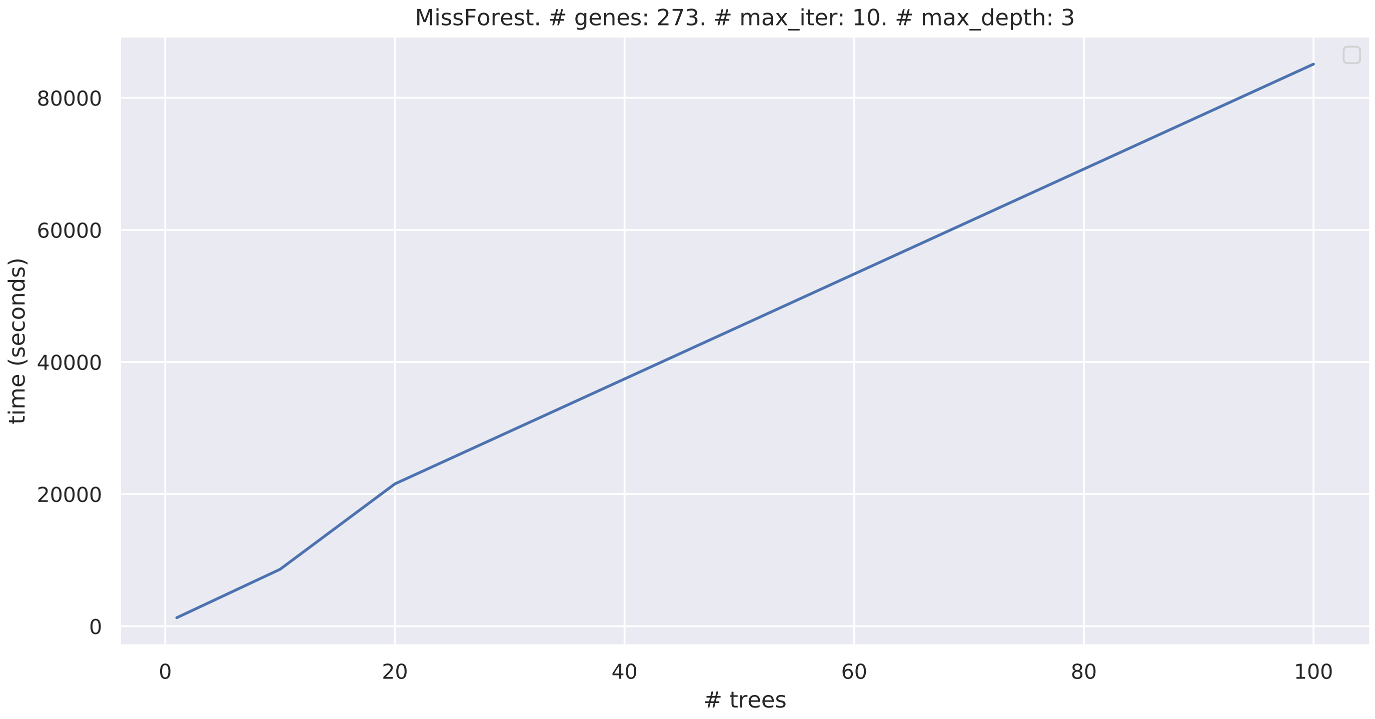

Figure S3: Runtime of MissForest algorithm (Stekhoven and Bühlmann, 2012) as we vary the number of trees. We ran the algorithm using all the samples on a subset of 273 trees from the Alzheimer's disease pathway.

3 SCALABILITY ANALYSIS FOR MICE

Figures S4 and S5 show the runtime of *a single* iteration of the MICE algorithm (Buuren and Groothuis-Oudshoorn, 2010) as we vary the number of genes and samples.

We kept all the non-specified hyperparameters to their default values. Our implementation is based on Python 3.7.6 and the library `sklearn` (Pedregosa et al., 2011), in particular `sklearn.impute.IterativeImputer`.

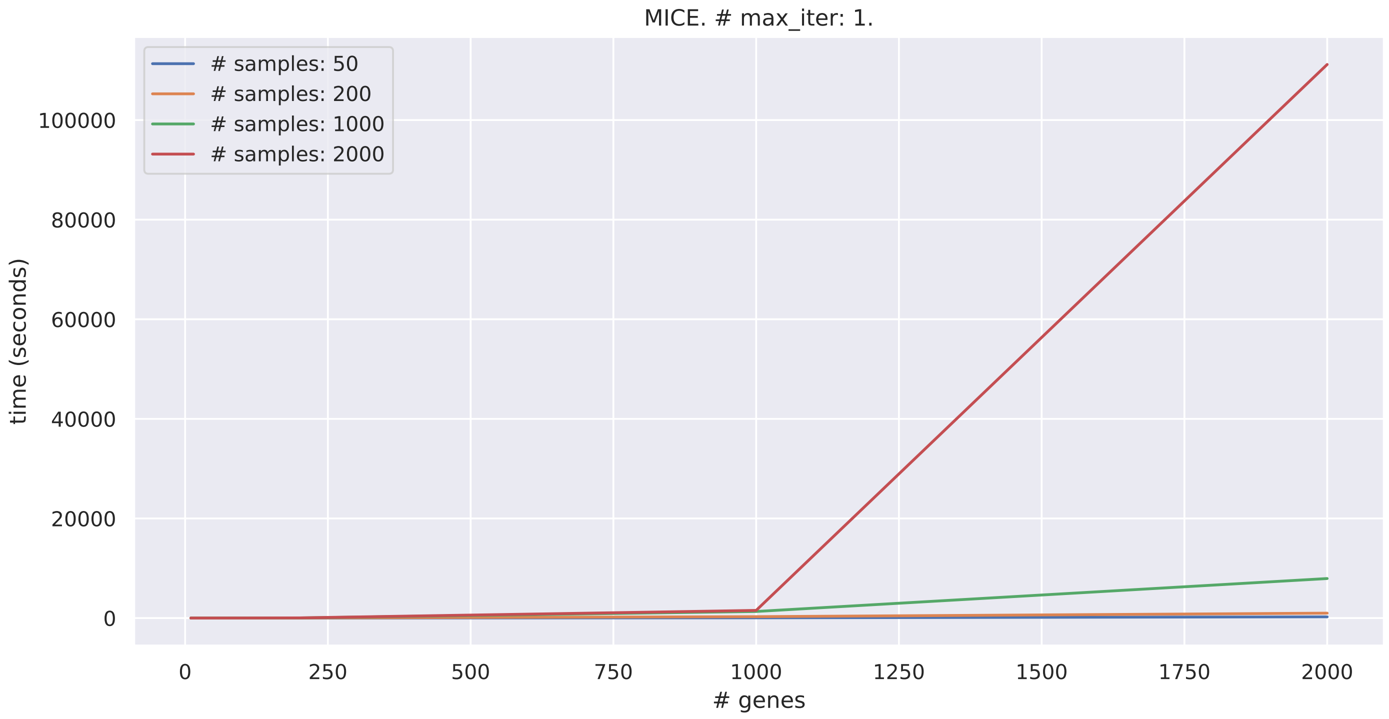

Figure S4: Runtime of *a single* iteration of the MICE algorithm (Buuren and Groothuis-Oudshoorn, 2010) as we vary the number of genes.

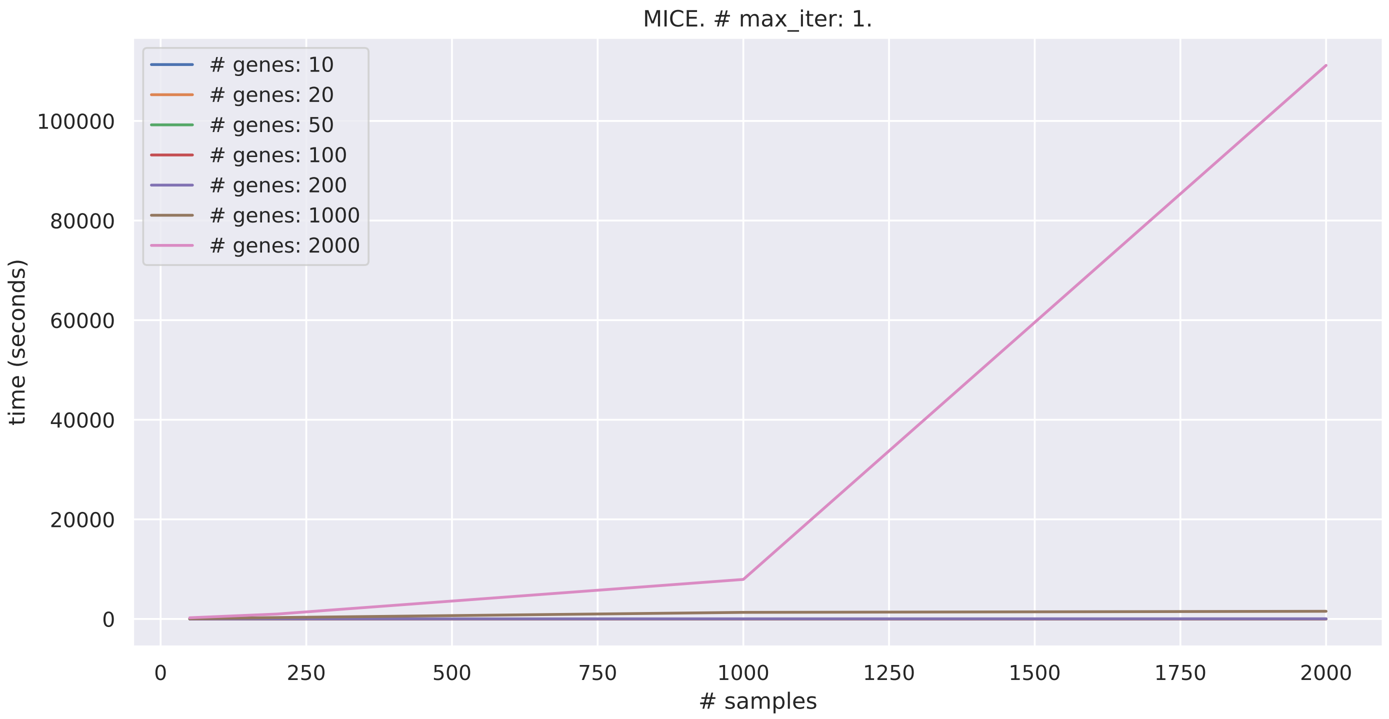

Figure S5: Runtime of *a single* iteration of the MICE algorithm (Buuren and Groothuis-Oudshoorn, 2010) as we vary the number of samples.

4 PMI HYPERPARAMETERS

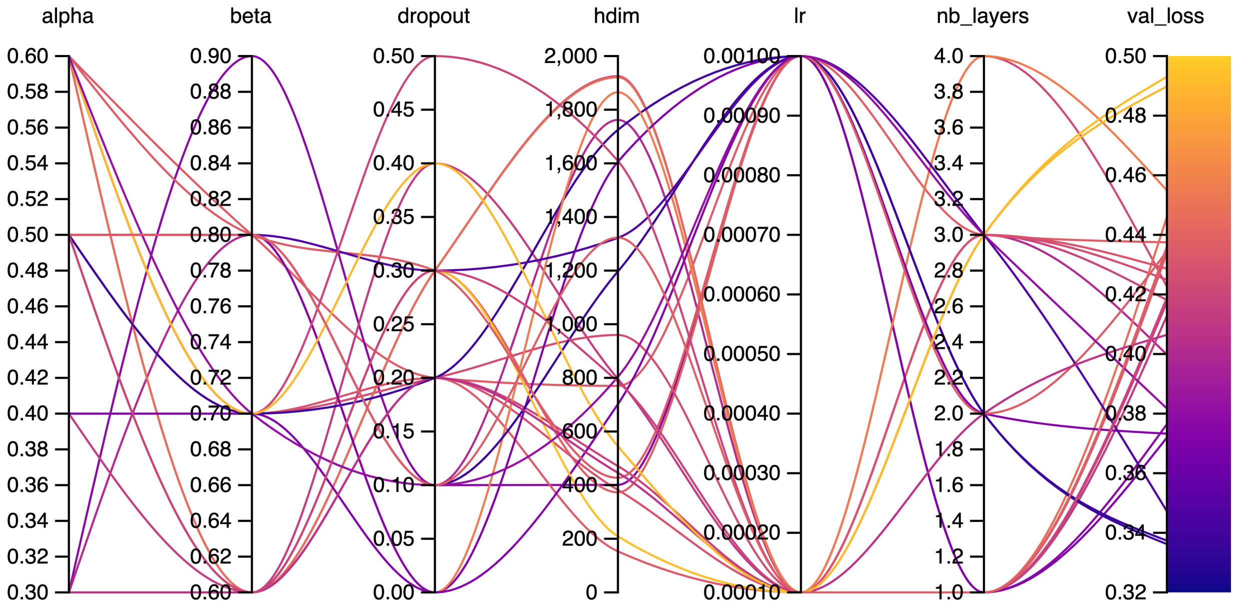

Figure S6: Exploration of the hyperparameter space for PIM on the subset of genes from the Alzheimer's disease pathway (*in-place mode*). The score axis shows the mean squared error on an independent validation set.

Figure S6 shows the validation MSE for different configurations of hyperparameters of PMI. We optimise the model using `wandb` (Biewald, 2020). We report the selected hyperparameters for each scenario in the following table:

	**All genes**		**Alzheimer**	
**PMI**	In-place	Inductive	In-place	Inductive
Alpha α	0.5	0.5	0.6	0.5
Beta β	0.9	0.5	0.9	0.5
Learning rate	0.0001	0.0001	0.001	0.001
Dropout probability	0	0.2	0	0.2
Number of layers	2	1	3	2
Hidden dimensionality per-layer	1366	3072	1383	1531

5 GAIN HYPERPARAMETERS

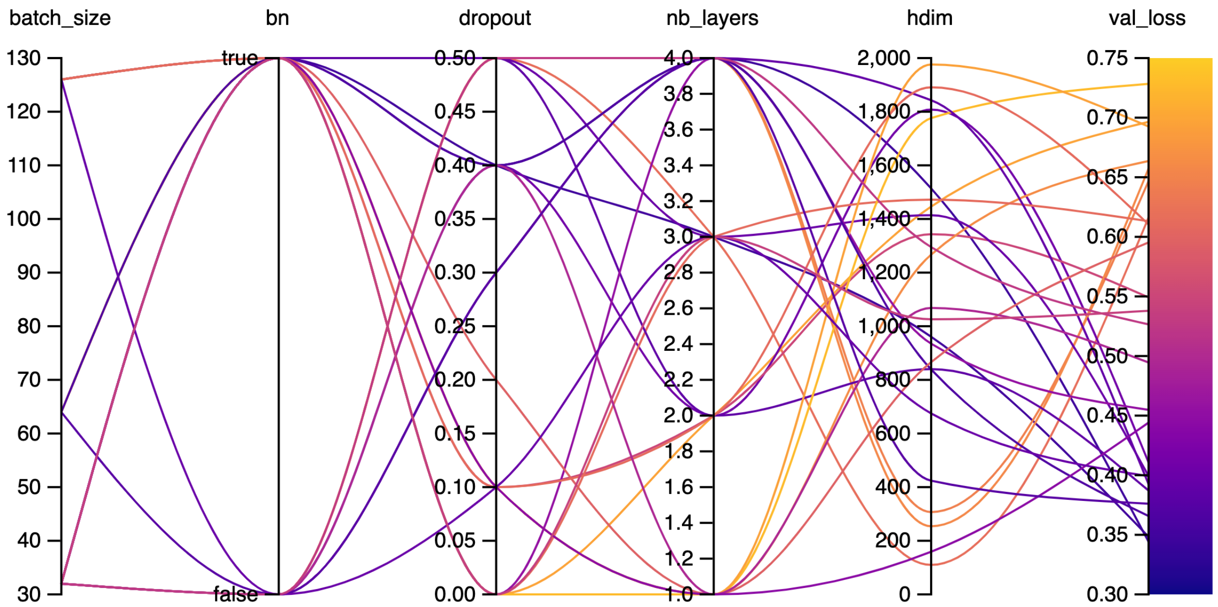

Figure S7: Exploration of the hyperparameter space for GTEx-GAIN on the subset of genes from the Alzheimer's disease pathway (*inductive mode*). The score axis shows the mean squared error on an independent validation set. In our experimentation we note that the model is fairly sensitive to the dimensionality of the hidden layers. On one hand, a small value leads to underfitting. On the other hand, a large value allows the model to trivially *copy-paste* the expression of the observed components.

Figure S7 shows the validation MSE for different configurations of hyperparameters of GAIN-GTEx. We optimise the model using `wandb` (Biewald, 2020). We report the selected hyperparameters for each scenario in the following table:

	**All genes**		**Alzheimer**	
**GAIN-GTEx**	In-place	Inductive	In-place	Inductive
Learning rate	0.001	0.001	0.001	0.001
Dropout probability	0	0.2	0.4	0.4
Number of layers	1	4	3	3
Hidden dimensionality per-layer	2403	1902	296	296

Regarding the output activation of GAIN, we leverage a linear and a sigmoid activation functions for the generator and discriminator, respectively. The linear activation ensures that the range of the output expression is unrestricted. We model both the generator and discriminator as MLPs with 4 hidden layers (2403 units each). In terms of the hyperparameter λ to trade off the adversarial and reconstruction losses of the generator (see Equation 13), we find that setting $\lambda = 1$ yields good results in all settings.

Mask and hint generation. At training time, for each training example, we sample the mask vector $\mathbf{m}$ from a Bernoulli distribution $B(1, p)$ parameterised by a random probability $p = 0.5$. To generate the hint vector $\mathbf{h}$ (see Equation 11), we sample $\mathbf{b}$ from $B(1, p)$, where $p = 0.5$.

6 ADDITIONAL FIGURES

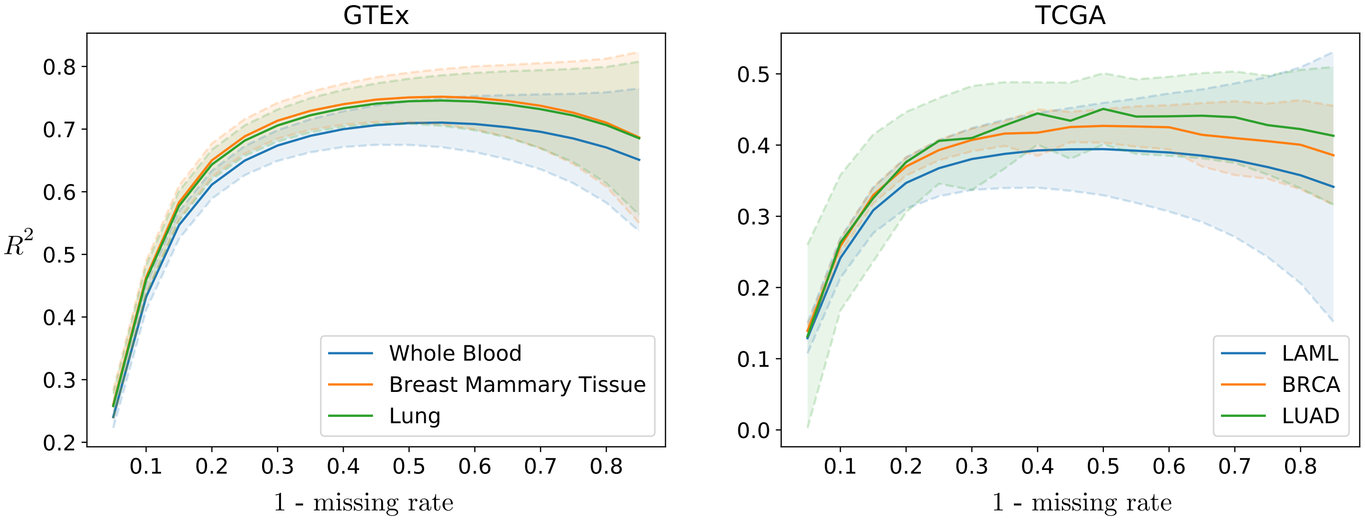

Figure S8: PMI R^2 imputation scores per tissue across missing rate for 3 TCGA cancer types and their healthy counterpart in GTEx. The shaded area represents one standard deviation of the per-gene R^2 scores in the corresponding tissue. The greater the rate of missingness, the lower the performance.

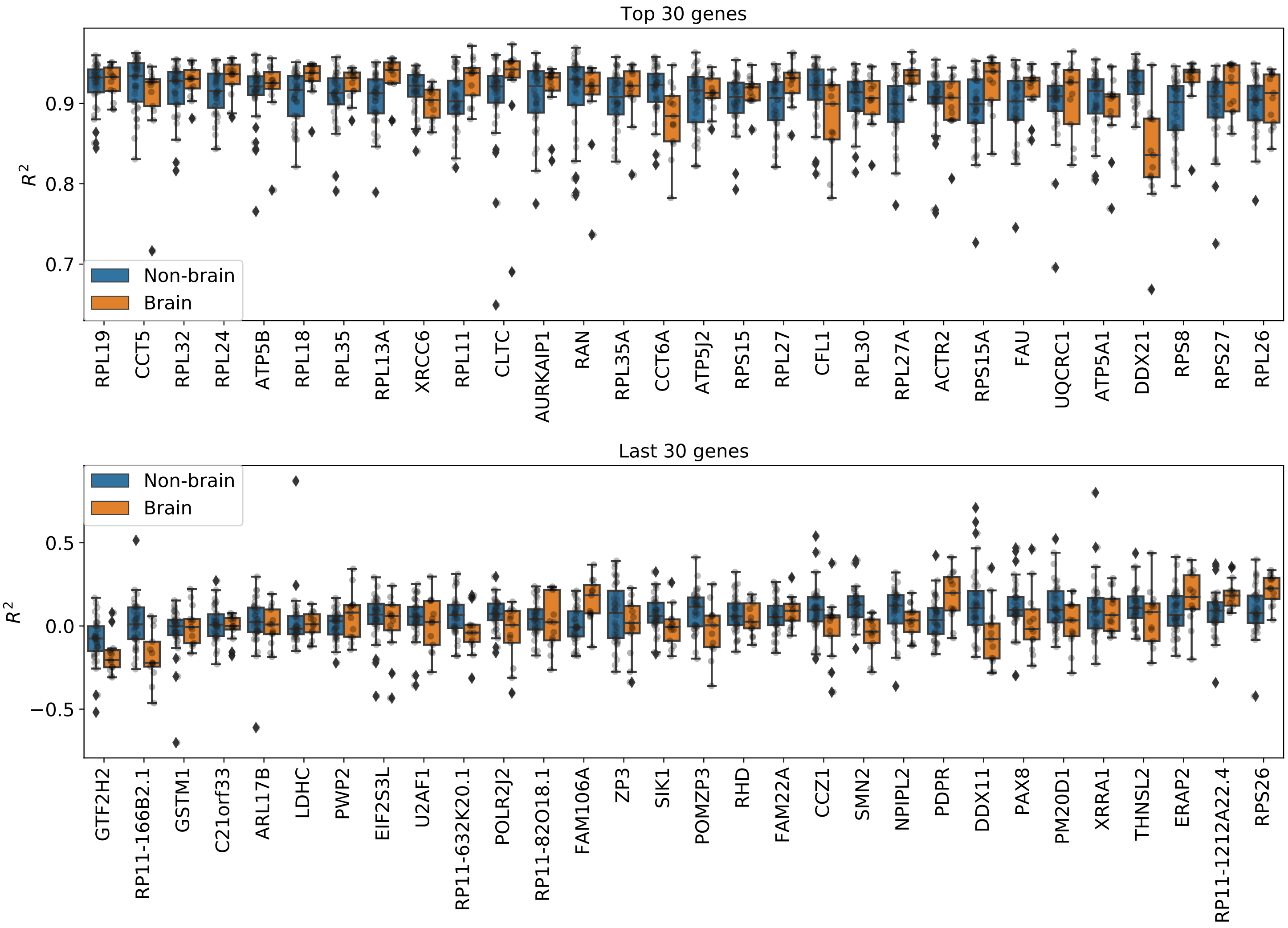

Figure S9: Per-gene imputation R^2 scores. We rank all the genes according to the average R^2 imputation scores across tissue types. We select the top 30 and last 30 genes. Interestingly, most of the best imputed genes are RPLs (L ribosomal proteins), which are known to be well conserved both evolutionarily and across tissue types.

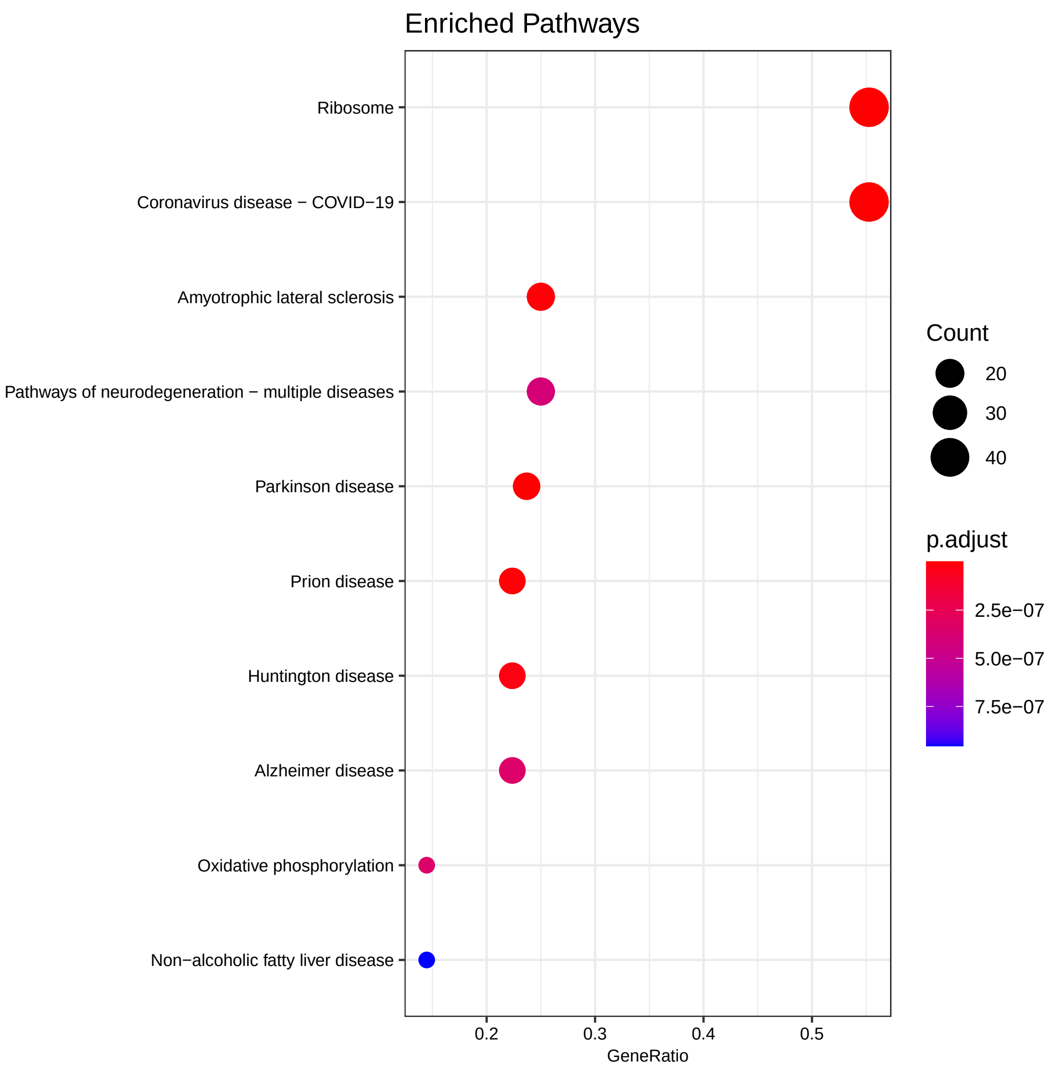

Figure S10: Top enriched KEGG pathways for overrepresentation analysis of the top 100 best imputed genes. Interestingly, we note that most of the best imputed genes are RPLs (L ribosomal proteins), which are generally well conserved evolutionarily and across tissue types.

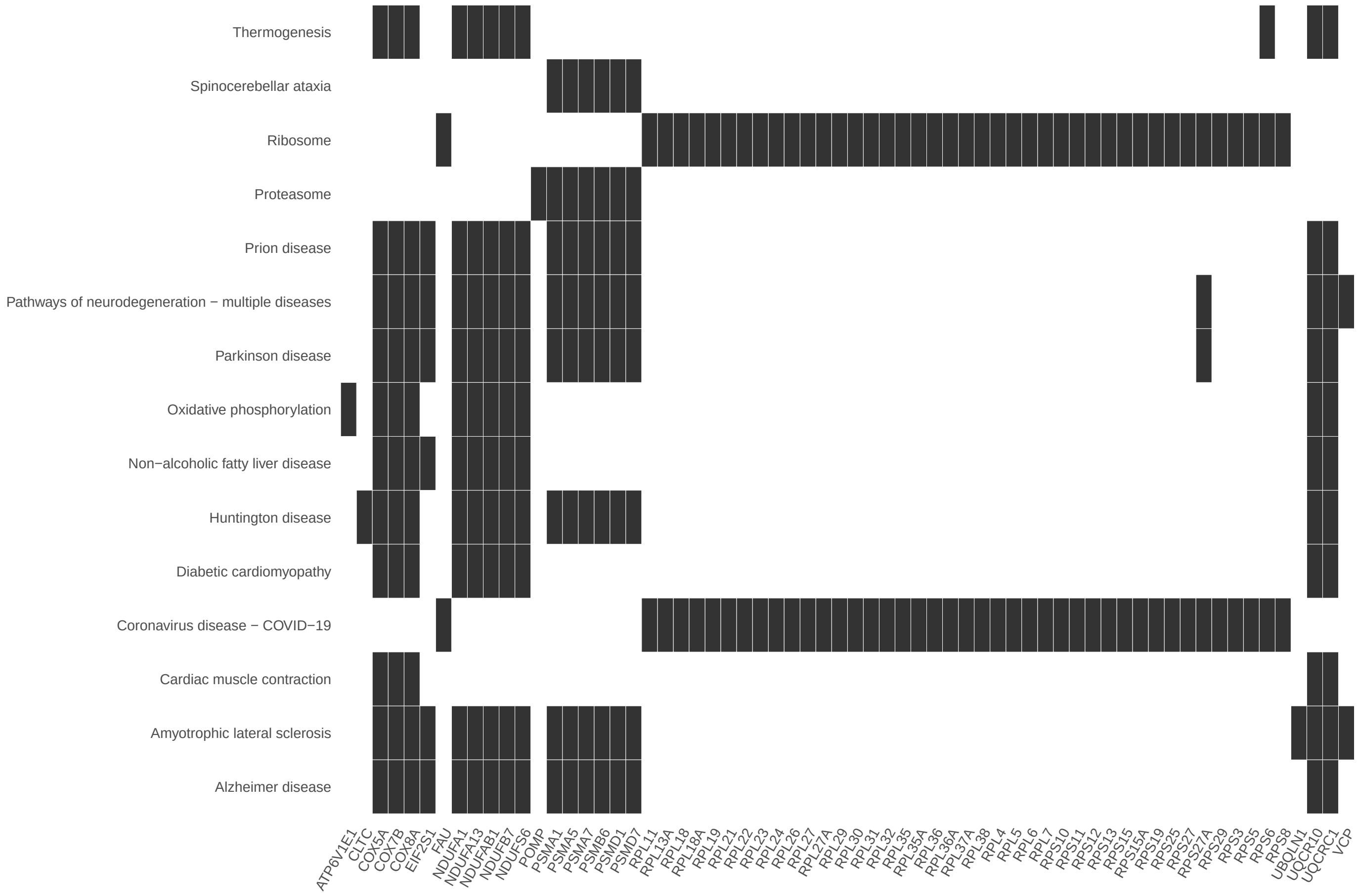

Figure S11: Heatmap of the gene-pathway associations for the top 100 imputed genes and the enriched KEGG pathways. Interestingly, we note that most of the best imputed genes are RPLs (L ribosomal proteins), which are generally well conserved evolutionarily and across tissue types.

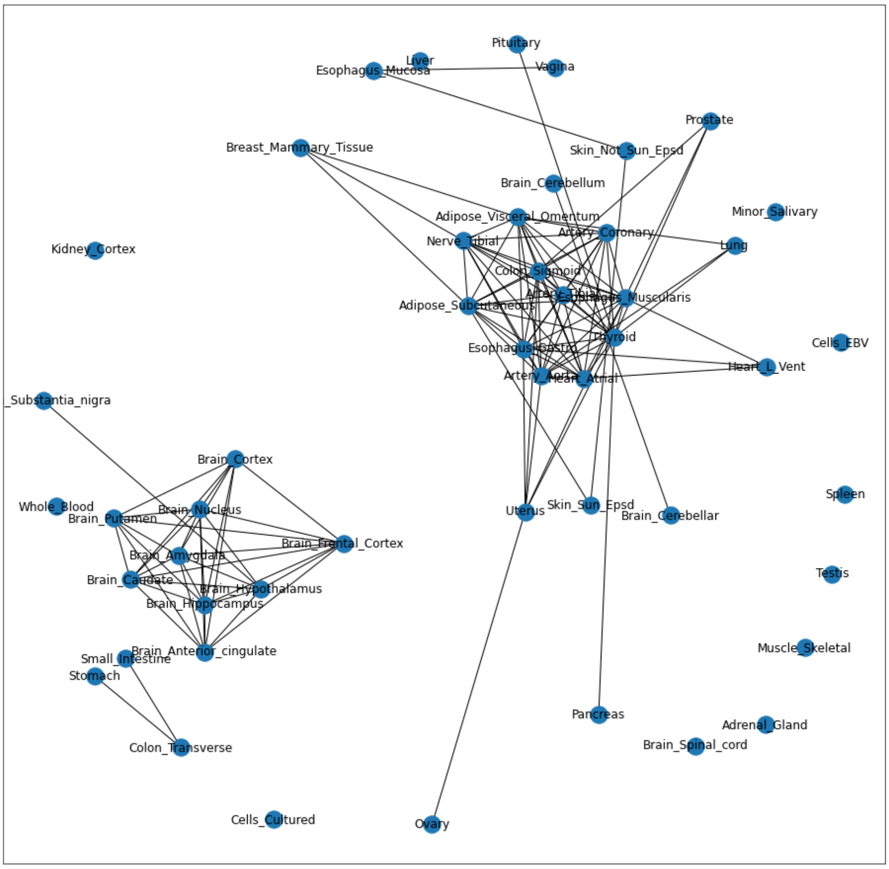

Figure S12: Network generated from the per-tissue R^2 scores (PMI; Alzheimer Pathway). For each pair of tissue types, we compute the Pearson's correlation coefficient between the tissue-specific vectors of per-gene R^2 scores. We then filter out the edges whose correlation is lower than an arbitrary threshold. This plot shows that the R^2 scores carry information about the tissue type and that the same genes in similar tissue types have similar R^2 scores.

REFERENCES

[Dataset] Biewald, L. (2020). Experiment tracking with weights and biases. Software available from wandb.com

Buuren, S. v. and Groothuis-Oudshoorn, K. (2010). mice: Multivariate imputation by chained equations in r. *Journal of statistical software* , 1–68

Pedregosa, F., Varoquaux, G., Gramfort, A., Michel, V., Thirion, B., Grisel, O., et al. (2011). Scikit-learn: Machine learning in Python. *Journal of Machine Learning Research* 12, 2825–2830

Stekhoven, D. J. and Bühlmann, P. (2012). Missforest—non-parametric missing value imputation for mixed-type data. *Bioinformatics* 28, 112–118

Yoon, J., Jordon, J., and Van Der Schaar, M. (2018). GAIN: Missing data imputation using generative adversarial nets. *arXiv preprint arXiv:1806.02920*
